# Triclosan and triclocarban exposure, infectious disease symptoms and antibiotic prescription in infants—A community-based randomized intervention

**DOI:** 10.1371/journal.pone.0199298

**Published:** 2018-06-28

**Authors:** Catherine Ley, Vandana Sundaram, Maria de la Luz Sanchez, Manisha Desai, Julie Parsonnet

**Affiliations:** 1 Division of Infectious Diseases, Department of Medicine, Stanford School of Medicine, Stanford, California, United States of America; 2 Quantitative Sciences Unit, Department of Medicine, Stanford School of Medicine, Stanford, California, United States of America; 3 Division of Epidemiology, Department of Health Research and Policy, Stanford School of Medicine, Stanford, California, United States of America; TNO, NETHERLANDS

## Abstract

**Background:**

Triclosan and triclocarban (TCs) are broad-spectrum antimicrobials that, until recently, were found in a wide variety of household and personal wash products. Popular with consumers, TCs have not been shown to protect against infectious diseases.

**Objectives:**

To determine whether use of TC-containing wash products reduces incidence of infection in children less than one year of age.

**Methods:**

Starting in 2011, we nested a randomized intervention of wash products with and without TCs within a multiethnic birth cohort. Maternal reports of infectious disease symptoms and antibiotic use were collected weekly by automated survey; household visits occurred every four months. Antibiotic prescriptions were identified by medical chart review. Urinary triclosan levels were measured in a participant subset. Differences by intervention group in reported infectious disease (primary outcome) and antibiotic use (secondary outcome) were assessed using mixed effects logistic regression and Fisher’s Exact tests, respectively.

**Results:**

Infectious illness occurred in 6% of weeks, with upper respiratory illness the predominant syndrome. Among 60 (45%) TC-exposed and 73 (55%) non-TC-exposed babies, infectious disease reports did not differ in frequency between groups (likelihood ratio test: p = 0.88). Medical visits with antibiotic prescriptions were less common in the TC group than in the non-TC group (7.8% vs. 16.6%, respectively; p = 0.02).

**Conclusions:**

Although randomization to TC-containing wash products was not associated with decreased infectious disease reports by mothers, TCs were associated with decreased antibiotic prescriptions, suggesting a benefit against bacterial infection. The recent removal of TCs from consumer wash products makes further elucidation of benefits and risks impracticable.

## Introduction

Triclocarban and triclosan (together, TCs) are broad-spectrum microbicides active against bacteria, fungi and some viruses [1–4]. Until recently, TCs were found in an ever-changing range of consumer products, including most notably bar soaps (triclocarban) and liquid soaps and toothpaste (triclosan). Introduced in the late 1950s, TCs rapidly became a near-ubiquitous human exposure: in 2011–2013, almost 37% and 72% of US human urine samples contained triclocarban and triclosan, respectively [5, 6]. Although presumably incorporated in wash products to prevent infection, there is no scientific evidence of TC efficacy in community settings. Whereas some studies indicate that the use of triclosan-containing toothpaste decreases plaque and gingivitis [7], use of TC-containing soaps in place of plain soaps has not been shown to decrease infectious illnesses in either households or pediatric populations [8–10].

In the environment, TCs are common contaminants. TCs have been found in 56–100% of surface water samples [11, 12] where adverse effects on aquatic microbiota have been of concern [13]. In experimental animal models, TCs have been reported to have endocrine effects [1]. Without evidence of benefit and with public concerns about environmental and physiological effects, TCs came under increasing scrutiny by regulatory agencies. They were banned from wash products in the European Union in 2010 and, in September 2016, the FDA similarly ruled that TCs can no longer be marketed in antimicrobial wash products. Other uses such as toothpaste, household plastics, industrial cleaning and hospital supplies were not included in this FDA ruling [14].

In 2011 we initiated a prospective birth cohort study, STanford’s Outcomes Research in Kids (STORK), to assess the effect of infectious diseases on infant growth and immune system development [15]. Within this cohort, we nested a randomized intervention of TC-containing wash products to determine whether TCs decreased infection during the babies’ first year.

## Materials and methods

### Recruitment, enrollment and follow-up

Detailed methods for the cohort have been described previously [15]. In brief, STORK participants were recruited through attendance at public obstetric clinics (Lucile Packard Children’s Hospital (Stanford, CA); Valley Health Center at Tully (Santa Clara Valley Medical Center [SCVMC], San Jose, CA) or by self-referral. An interested participant was eligible if: fluent in English or Spanish; aged 18–42 years; within 20 weeks gestation of a low-risk pregnancy with a single fetus; not known to have non-gestational diabetes or other endocrine conditions; willing to provide biological samples from and access to medical records for herself and her infant; and intending to remain in the San Francisco Bay Area for the subsequent 15 months. Written informed consent was administered at a baseline home visit convenient to the mother; written parental/guardian informed consent for the baby’s participation was obtained once the baby was born.

Mothers were invited at cohort enrollment to participate in a randomized parallel intervention of the effect of TC-containing wash products on infectious disease in the baby. We assumed that a total of 63 babies in each group would be sufficient to detect a 20% decrease in illness days due to TC exposure, assuming a prevalence of 40 days of illness per year, an alpha of .05 and power of 80%. Blocked randomization used the biased coin method so that intervention groups had an equal proportion of first-born infants from each enrollment site (block size = 4). The random allocation sequence was generated in collaboration with the Stanford Center for Clinical and Translational Research and Education (SPECTRUM); it was implemented using sequentially numbered lists, one for each group; participants were enrolled by the STORK study coordinator and assigned to their allocated group by the STORK data manager. Consented participants selected wash products for their household (liquid and bar soap, toothpaste, dishwashing liquid) from one of two lists of commercial products, all either containing or not containing TCs according to randomization group. Instructions included only that products be used in the household (including on the upcoming baby) as desired (i.e., used as in the “real world”). Supplies were replenished every four months. Toothpastes were not provided to households until after the baby was born; until then, mothers could use any product they chose. In non-TC households, with the exception of toothpaste prior to the baby’s birth, TC-containing products were replaced by study-provided non-TC-containing products at enrollment but not at later visits.

Information collected at the baseline household visit included demographic and household characteristics, the mother’s use of cleaning products or chemicals at her workplaces and bathing habits [15]. The interviewer provided an assessment of household cleanliness using a modified version of the Environmental Cleanliness and Clutter Scale [16]. Additional information was ascertained at each subsequent household visit, which occurred approximately every four months after enrollment.

A short automated survey was administered every week during the remainder of the pregnancy by telephone or email to ascertain infectious disease symptoms, their duration, and antibiotic use, or, if none were reported, details on recent behaviors (sleep, stress and physical activity) [15]. After delivery, the weekly automated survey pertained to the baby’s health (if ill: infectious disease symptoms, their duration, visits to a health care provider (HCP) and antibiotic use, or if well: breastfeeding status, introduction to new foods, sleep).

Compensation for participation included, besides free wash products, a $10 gift card per participant per household visit and a $25 gift card for each 16 weekly surveys completed (corresponding approximately to every four-monthly household visit). The study was approved by Stanford University’s Administrative Panel on Human Subjects in Medical Research and the SCVMC Institutional Review Board (ClinicalTrials.gov identifier: NCT01442701) and supported by the National Institutes of Health [5R01HD063142 and 5R21ES023371].

Chart review from any available medical records from visits for illness from the baby’s first year of life was performed to determine oral antibiotic prescriptions and their associated diagnoses; associated diagnoses were classified as either “definite” bacterial infection (e.g., abscess, cellulitis) or “possible” bacterial infection (e.g., conjunctivitis, pneumonia, otitis media). Abstractors were blinded as to each baby’s intervention group assignment.

All questionnaire information was collected electronically offline by STORK study staff during a household visit. Weekly automated surveys were administered using Precision Polling (Palo Alto, CA) if by telephone or Qualtrics (Provo, UT) if by email. Medical record information was collected in Medrio (San Francisco, CA). All data were managed in REDCap [17] hosted at Stanford University.

### Urinary triclosan measurements

Urine specimens from a random sample of mothers and babies collected at the second household visit after the baby’s birth (at approximately seven months of age) were tested for triclosan. (Urinary triclocarban levels were not assessed.) Triclosan levels were measured using liquid chromatography-mass spectrometry with liquid-liquid extraction using ethyl acetate (Supporting Information—S1 Protocol). Testing was performed at the Vincent Coates Foundation Mass Spectrometry Laboratory, Stanford University Mass Spectrometry (http://mass-spec.stanford.edu) (Stanford, CA).

The values of the first and the fourth quartiles of the distribution of all available maternal urinary triclosan levels, 13.6 pg/μl and 334 pg/μl, were used to define discrete triclosan exposure measures (see below).

### Statistical methods

#### Key outcomes

Our primary outcome was the presence of infectious disease symptoms in babies by week. Infectious disease symptoms were counted as present if the mother reported at least one symptom during the weekly survey, and as absent if no symptoms were reported; if the survey was not completed, the week counted as missing.

Secondary outcomes included both the proportion of weeks with illness in mothers and the same in babies. Proportion of weeks with illness was defined as “sick-weeks” divided by response weeks, where sick-weeks included all weeks of follow-up where a participant reported at least one day of infectious disease symptoms (upper respiratory infection [URI: cough and/or cold (and/or ear-pulling in infants)]; fever; vomiting; and/or diarrhea) and response weeks were the total number of weeks for that participant in which a survey was completed. Additional outcomes included the use of antibiotics either reported (from the weekly survey) or identified by prescription with medial record review.

#### Key exposure and co-variables

The primary exposure of interest was household intervention group assignment. Season was defined as winter (October-March) or summer (April-September), based on the date of the weekly survey.

#### Intent-to-treat and per-protocol populations

We conducted two primary analyses: a modified intention-to-treat (mITT) analysis and a modified per-protocol (mPP) analysis. The mITT was comprised of all households with at least one completed survey (i.e., one record of presence or absence of infectious disease symptoms) for the baby. The mPP included a subset of all households where a maternal post-enrollment urinary triclosan measure was available, with households categorized into one of two groups according to this maternal urinary triclosan level: households were considered as TC-exposed if the triclosan level exceeded 334 pg/μl, and as non-TC-exposed if the triclosan level was less than 13.6 pg/μl (households with triclosan levels in the middle two quartiles were excluded from consideration).

#### Descriptive statistics

We tested differences between intervention groups using Wilcoxon rank-sum tests or t-tests for continuous variables and Wald chi-square or Fisher’s exact tests for categorical variables. We assessed the correlation between mother and baby urinary triclosan levels using the Spearman correlation coefficient and between the randomized intervention group and the group assigned based on urinary triclosan levels using the Phi coefficient.

#### Primary analyses

We used mixed effects logistic regression techniques to test for differences in presence of infectious disease symptoms in babies by intervention group over time (Supporting Information–S1 Text). We ran our analysis using the SAS procedure Proc Glimmix and specified an unstructured correlation structure. We ran a full model and a nested model excluding interaction terms of group by time and conducted a likelihood ratio test to compare the two models.

#### Secondary analyses

To identify a difference in infection over time in the highest vs. the lowest TC exposure groups (mPP cohort), we compared households in the first quartile of maternal urinary triclosan level (<13.6 pg/μl) to those in the fourth quartile (≥ 334 pg/μl).

All analyses were performed in SAS Version 9.4 (Cary, NC) or R [18].

## Results

**Fig 1:**
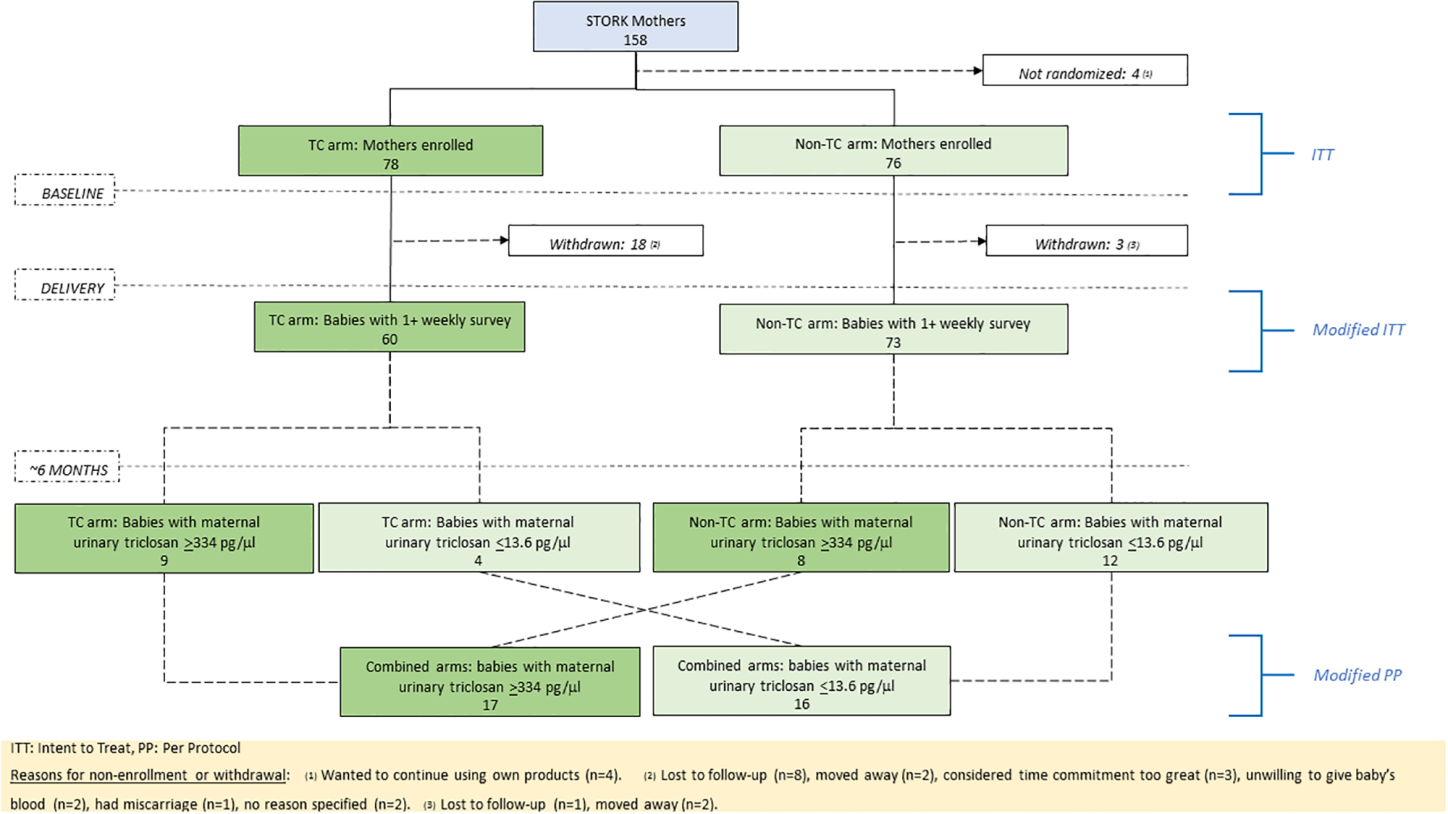
Participant disposition by randomization group, showing modified intent-to-treat (mITT) and modified per-protocol (mPP) cohorts.

From July, 2011 to February, 2015, 158 mothers were enrolled in the STORK cohort, of whom 154 (97%) participated in the TC intervention (78 (51%) randomized to the TC group and 76 (49%) to the non-TC group; Fig 1). The reason for non-participation in the intervention was the desire to continue use of their current wash products (4/4, 100%). At enrollment into the intervention, approximately one third of mothers were expecting a first child; their average age was 30 years, 64% were Hispanic, and 59% were foreign-born (Table 1). Households contained a median of four residents, of whom approximately two were children aged less than 18 years; households were clean but overcrowded [19] with a median of 1.2 people per room. Maternal exposure to cleaning products and chemicals outside of the home was quite common (22%) and most mothers bathed at least once daily (88%). No differences in any of these characteristics were observed between intervention groups.

**Table 1:** Maternal and household characteristics at enrollment, by intervention group (ITT cohort^a^).

	TC Group (n = 78)	Non-TC Group (n = 76)	Total (N = 154)
First-born child	26 (33%)	23 (30%)	49 (32%)
Maternal age at enrollment (years)			
[mean (SD)]	29 (6.0)	31 (6.3)	30 (6.2)
[median (IQR)]	30 (24–34)	31 (26–35)	31 (25–35)
Maternal race/ethnicity			
Hispanic	54 (69%)	45 (59%)	99 (64%)
White non-Hispanic	11 (14%)	13 (17%)	24 (16%)
Black non-Hispanic	4 (5%)	3 (4%)	7 (5%)
Asian	6 (8%)	12 (16%)	18 (12%)
Not specified	3 (4%)	3 (4%)	6 (4%)
Mother’s country of birth			
U.S.	30 (38%)	33 (43%)	63 (41%)
Other	48 (62%)	43 (57%)	91 (59%)
Maternal education level			
Less than high school	23 (29%)	22 (29%)	45 (29%)
High school	18 (23%)	15 (20%)	33 (21%)
Some college	27 (35%)	17 (22%)	44 (29%)
Post college	10 (13%)	22 (29%)	32 (21%)
Household size [median (IQR)]	4 (3–6)	4 (3–6)	4 (3–6)
Household with minors (age <18 years)	61 (78%)	57 (75%)	118 (77%)
Minors in household at enrollment			
[mean (SD)]	2.0 (1.2)	1.9 (1.1)	2.0 (1.1)
[median (IQR)]	2 (1–3)	2 (1–3)	2.0 (1–3)
Household cleanliness score^b^ [median (IQR)]	4 (1–5)	3 (1–5)	4 (1–5)
Crowding (people/room)			
[mean (SD)]	1.5 (1.0)	1.3 (0.8)	1.4 (0.9)
[median (IQR)]	1.3 (0.8–2.0)	1.2 (0.8–1.5)	1.2 (0.8–1.8)
Use of cleaning products or chemicals at work			
Yes	16 (21%)	18 (24%)	34 (22%)
No	33 (42%)	31 (41%)	64 (42%)
Not applicable	29 (37%)	27 (36%)	56 (36%)
Bathing habits			
More than once per day	10 (13%)	13 (17%)	23 (15%)
Daily	57 (73%)	55 (72%)	112 (73%)
Every other day	11 (14%)	8 (11%)	19 (12%)

ITT: Intent to treat; IQR: interquartile range; SD: standard deviation; TC: triclosan/triclocarban

^a^ For maternal and household characteristics at enrollment by intervention group in the mITT cohort, see Supporting Information–S1 Table.

^b^ Household cleanliness score (0–10), where 0 = very clean; assessed by interviewer, summed over four subscales (A-D). A: Floor and carpets 0: very clean; 1: acceptably clean; 2: looks as if not cleaned or swept for days; scattered rubbish; 3: very dirty and looks as if not cleaned for months. B: Walls and visible furniture and windowsills 0: very clean; 1: acceptable/reasonably clean; 2: dusty and dirty surfaces (dirt comes off with finger); 3: grime or dirt on walls (greasy, messy, wet and/or grubby furniture). C: Bathroom and toilet 0: very clean; 1: acceptable/reasonably clean; 2: untidy, unclean/grubby surfaces; 3: dirty surfaces with scattered rubbish; faces or urine on outside of toilet bowl. D: Kitchen 0: very clean; 1: acceptable/reasonably clean; 2: untidy with dirty surfaces, rubbish mainly in garbage bin; 3: surfaces dirty, piles of unwashed dishes, bins overflowing, rotten or moldy food.

Follow-up of mothers during pregnancy was similar for both intervention groups (median proportion of weekly surveys completed from enrollment to delivery: 89% for both TC and non-TC groups, p = 0.75). Loss to follow-up or withdrawal from the study prior to the baby’s birth (n = 21) was more common overall in the TC than in the non-TC group (18 (23%) vs. 3 (4%), p<0.001; Fig 1). Nine mothers could not be contacted and were lost to follow-up; for the remaining 12 mothers, reasons for withdrawal included: a move away from the area (n = 4), time commitment perceived as too great (n = 3), unwillingness to provide blood from the baby (n = 2), a miscarriage (n = 1) and no specific reason (n = 2); no provided reason for withdrawal appeared related to the intervention.

Totals of 60 and 73 babies providing at least one weekly survey were born into the TC and non-TC groups respectively, constituting the analysis sample (mITT; Supporting Information–S1 Table). Both follow-up time and proportion of households withdrawn from the study were similar between groups (Table 2). Babies in the two groups were equally likely to be breastfed and for the same amount of time. Use of day care also was not different between intervention groups. Among babies, infectious disease symptoms were more common in winter months and with increasing age; babies younger than 80 days (~2.6 months) of age rarely experienced infectious diseases symptoms (Supporting Information–S1 Fig).

**Table 2:** Follow-up, infection and antibiotic use, by intervention group (mITT cohort).

	TC Group (N = 60)	Non-TC Group (N = 73)	p-value^a^
Follow-up time in babies (weeks) [median (IQR)]			
Overall	51 (50–51)	51 (49–51)	0.62
Non-withdrawals	51 (51–51)	51 (51–51)	0.59
Withdrawals	27 (13–51)	48 (26–51)	0.79
Withdrawn before 1 year of follow-up	15 (25%)	23 (32%)	0.41
Breast feeding (any vs. none)	59 (98%)	72 (98%)	1.0
Weeks of breastfeeding during the first year [median (IQR)]	46 (20–49)	47 (15–49)	0.98
Use of day care during the first year	8 (13%)	11 (15%)	0.78
Percent surveys completed [median (IQR)]			
Overall	84 (75–91)	84 (65–90)	0.62
Non-withdrawals	88 (82–92)	88 (79–92)	0.59
Withdrawals	77 (51–85)	69 (51–84)	0.48
Urinary triclosan level at Visit 2^b^ in pg/μl [median (IQR)]			
Mother (TC group: n = 28; non-TC group: n = 37)	90.9 (24.5–788.4)	58.1 (10.4–268.0)	0.10
Baby (TC group: n = 23; non-TC group: n = 17)	6.3 (0–32.7)	3.0 (0–7.8)	0.20
Proportion of weeks with illness in pregnant women (unadjusted)			
[mean (SD)]	0.06 (0.11)	0.05 (0.08)	ND
[median (IQR)]	0.01 (0–0.07)	0.02 (0–0.09)	0.53
If sick, proportion of sick weeks [median (IQR)] in pregnant women with:			
URI	0.7 (0–1)	0 (0–1)	0.11
Diarrhea	0 (0–0.02)	0 (0–0.14)	0.32
Fever	0 (0)	0 (0–0.21)	0.10
Vomiting	0 (0–0.07)	0 (0–0.04)	0.86
Proportion of weeks with illness in babies (unadjusted) *			
[mean (SD)]	0.07 (0.09)	0.1 (0.12)	ND
[median (IQR)]	0.05 (0.02–0.1)	0.06 (0.02–0.12)	0.16
If sick, proportion of sick weeks [median (IQR)] in babies with:			
URI	0.86 (0.76–0.99)	0.91 (0.76–1.0)	0.51
Diarrhea	0.05 (0–0.24)	0.05 (0–0.22)	0.79
Fever	0.2 (0.08–0.3)	0.1 (0.05–0.26	0.18
Vomiting	0 (0–0.06)	0.02 (0–0.11)	0.12
Antibiotic use			
Visits ^c^ where an antibiotic was prescribed for possible/definite bacterial infection	12 (7.9%)	31 (16.6%)	0.02
Babies ^d^ prescribed an antibiotic for any indication	10 (23.3%)	17 (34.0%)	0.26
Reported antibiotic use by weekly survey	24 (40.0%)	30 (41.1%)	0.90

mITT: modified intent to treat; ND: not done; IQR: interquartile range; SD; standard deviation; TC: triclosan/triclocarban; URI: upper respiratory infection (cold and/or cough [and/or ear pulling in babies]).

^a^ Wilcoxon rank-sum test or t-test for continuous variables; Wald chi-square or Fisher’s exact test for categorical variables

^b^ Baby aged approximately 7 months (mean: 6.7; SD = 1.1)

* Secondary endpoint. (See text for the primary endpoint [odds of infection across time] where p = 0.88 from mixed effects logistic regression modeling.)

^c^ Chart review was performed for 339 medical visits (152 (44.8%) in the TC group and 187 (55.2%) in the non-TC group)

^d^ 93 babies had available medical charts (43 (46.2%) in the TC group and 50 (53.8%) in the non-TC group)

### Urinary triclosan levels

Urinary triclosan levels at the second household visit after delivery (when the baby was approximately seven months old) were available for 65 mothers (42%) and 40 babies (30%) (Table 2). Among mothers, 26/28 (93%) in the TC group and 32/37 (86%) in the non-TC group had detectable (non-zero) urinary triclosan levels; median urinary triclosan levels were somewhat higher in the TC- than in the non-TC group (91 vs. 58 pg/μl; p = 0.10). Based on the 334 pg/μl cut-off for high exposure (i.e., top quartile of urinary triclosan), nine of 13 mothers (69%) assigned to the TC group were heavily exposed to TC compared to eight of 20 mothers (40%) assigned to the non-TC group (Phi coefficient = 0.29; Fig 1). Urinary triclosan levels in babies were extremely low compared to those in mothers, with median levels similar between intervention groups (6 vs. 3 pg/μl, p = 0.21) (Table 2).

### Primary outcome

**Fig 2:**
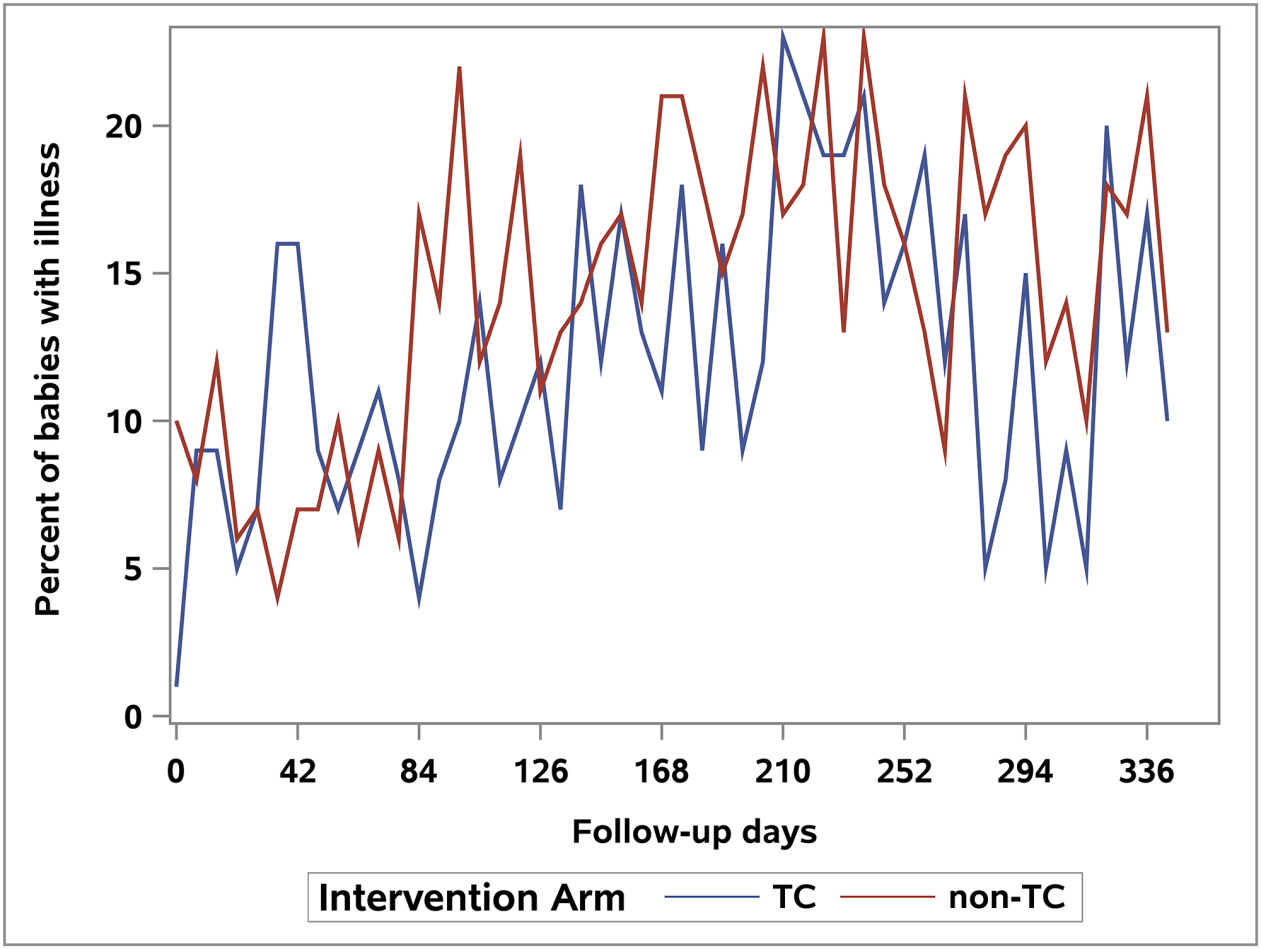
Proportion of babies reporting infectious disease symptoms by week over the first year (mITT cohort).

Logistic regression modeling did not identify a significant difference in the odds of infectious illness symptoms over time in babies by intervention group in the mITT cohort (likelihood ratio test: mITT analysis, p = 0.88, Fig 2). An analysis excluding the first 90 days after birth (when few illnesses were reported) showed similar findings (p = 0.68). The mPP analysis comparing babies of mothers in the first and fourth quartiles of urinary triclosan also did not show any difference in the odds of illness over time (p = 0.0.83; Supporting Information—S2 Fig).

### Secondary outcomes

Reported illnesses in the pregnant mothers were few (Table 2). Among these mothers, the proportion of weeks with illness was not different between groups. Among pregnant mothers who did report illness, the proportions of follow-up weeks with URI, diarrhea, fever and/or vomiting did not differ significantly between groups (Table 2).

Among babies, the proportion of weeks with illness was similar between intervention groups (Table 2). Among sick babies, the proportions of follow-up weeks with URI, fever, vomiting or diarrhea did not differ between groups.

A total of 93 babies had at least one diagnosis of illness with an associated medical record available for review, for a total of 339 medical care visits. The proportion of medical care visits where antibiotics were prescribed for possible or definite bacterial infections (43 visits in total) was lower in the TC group compared to the non-TC group (7.8% vs. 16.6%, p = 0.02). Indications for antibiotic prescription included otitis media (60.5%), upper respiratory infection (25.6%), conjunctivitis (18.6%) and miscellaneous other (41.9%) (these categories were not mutually exclusive), with similar distributions between groups. Overall, however, the proportion of babies who received at least one antibiotic prescription (for any indication) based on medical record review was not statistically different between the two groups (23.3% in the TC group vs. 34.0% in the non-TC group, p = 0.26; Table 2). Maternally reported use of antibiotics by weekly survey was not different between groups either overall (Table 2) or in instances of use per child (median number of weeks that use was reported [IQR]: 1 [0–2.5] in the TC group vs. 0 [0–3] in the non-TC group, p = 0.99).

## Discussion

In this study, randomization of households to “real world” use of wash products with TCs was not related to the occurrence of infectious illness in babies during their first year of life. These findings remained consistent even after taking into account both seasonality and the enrollment period, and after comparing only those babies whose mothers had the highest levels of urinary triclosan to those with mothers with the lowest. Our majority immigrant multiethnic families provided both weekly assessments of illness in their children and at household visits every four months; with this highly granular follow-up, this intervention comparing antimicrobial soaps to regular soaps in the community setting showed that TC-containing soaps did not prevent illness, consistent with prior literature [20].

Several caveats, however, must be noted. In this community setting of primarily underserved families, the rate of reported infections was low in both pregnant mothers and babies. When illnesses did occur, they were typically URIs, with few reported instances of fever, vomiting or diarrhea. Most URIs, however, are viral in etiology [21] and, although TCs are reported to have activity against some viral pathogens, the breadth of viral activity is not well defined. The expectation is that TCs would limit bacterial disease—a fact made evident by the “anti-bacterial” labeling on most TC products. It is notable, then, that children in the TC group received fewer prescriptions for courses of antibiotics than those in the non-TC group according to medical record review. This halving of documented antibiotic prescriptions in the TC group may signal some antimicrobial benefit of TCs with routine household use. Whether this putative benefit derives from killing of pathogens on surfaces or immunomodulatory effects in the host cannot be determined from our data. In both in vitro and animal models, TCs have been linked to a variety of immunologic consequences, both boosting and suppressive in nature [22–24]. However, the findings that TCs did not alter viral symptoms and that triclosan levels in infants were low suggest that alterations in host immunology are unlikely to have led to differences in antibiotic prescriptions.

Although the halving of antibiotic prescriptions in the TC group was intriguing, we could not confirm this finding by review of mothers’ responses to weekly surveys regarding antibiotic use. Half of babies were reported by their mother to have received antibiotics prior to their first birthday, a finding comparable to other studies [25]. Mothers, however, were often unclear as to what medicines were antibiotics: when a random subset of 60 episodes was investigated to confirm their reported antibiotic use, 37% of “antibiotics” were found to be non-antibiotic medications, primarily analgesics (acetaminophen (55%), ibuprofen (5%)) (unpublished data). Because of this inaccuracy, we relied on medical records to determine antibiotic prescription receipt. It should be noted, however, that this too could be inaccurate. Although we requested records from the babies’ primary health care facilities, many families may have visited other locations from which we did not receive records (e.g., urgent care and emergency rooms). There is little reason to suspect, however, that use of these outside facilities was related to randomization by study group, making underreporting of antibiotics from medical records an unlikely confounder.

TC exposure was common even in the non-TC group, as 86% of mothers tested had detectable urinary triclosan levels. While this study successfully randomized households to TC-containing wash products, we did not place restrictions on the use of non-study-provided wash products, and levels of triclosan overall were comparable to population-based studies [26, 27]. Moreover, TC-containing wash products in the home clearly are not the only source of personal TC exposure; indeed, one fifth of our mothers reported exposure to cleaning products or chemicals—possibly including TCs—at their workplaces. Such misclassification might mask a true association between TC exposure from wash products and illness. Additionally, despite the high TC levels in mothers, levels in babies in this study were extremely low. We believe it unlikely that TCs would directly decrease infection rates by altering the babies’ microbial flora. Although this possibility does exist [28], in adults, at much higher levels of exposure, TC-containing wash products have been found to have no effect on the microbiome of the gut or the mouth [29]. The use of TCs in household cleaning products, however, may have a substantial effect on environmental microbes, by decreasing their overall abundance and possibly restricting their variation [30]. Any impact of TCs on antibiotic use, then, would result from preventing transmission from contaminated surfaces that are cleaned with these wash products.

Several limitations are inherent in this study. First, the intervention was not blinded, with household members able to identify their exposure group by reading product labels. To verify exposure, we used spot measures of urinary triclosan that are known to correlate highly with 24-hour urine samples [31]. Nevertheless, although we found that TC-randomized mothers had higher urinary triclosan levels, they were not statistically significantly higher compared to the levels in non-TC-randomized mothers. Second, no exposure dose information was available. While TC is absorbed through the skin [32], triclosan-containing toothpaste is likely the main source of urinary triclosan in mothers; infants, however, are unlikely to be directly exposed and toothpaste use likely will not alter infection risk in children. The amount of exposure to triclosan in the household environment through wash products and dish soap use is not quantifiable. Third, not all infectious illnesses in the child were diagnosed by a HCP. Maternal report of illness, however,—particularly airway-related disease—has been shown to be highly valid [33]. Had we chosen to consider only diagnoses observed by a HCP, mild illnesses that were not brought to medical attention would have been missed. Mothers in each intervention group were equally likely to complete their weekly survey, minimizing the likelihood of differential recall bias. Finally, our analysis of antibiotic prescriptions was incomplete, as not all medical records were available for review.

In summary, using a randomized intervention nested within a well-characterized birth cohort, we found no evidence that “real-life” use of TC-containing wash products changed the occurrence of infectious disease in young children and their mothers. Considerable exposure to TCs from other sources and the preponderance of viral infection, however, may mitigate any effects of household exposure. The finding that children in TC-exposed households had half the antibiotic prescriptions of children in non-TC-exposed houses could indicate the potential of TCs having a large impact on antibiotic use in the US. Regulatory removal of TCs from household wash products, however, will make further investigation of TC effects on child health impracticable.

## Supporting information

S1 Table Maternal and household characteristics at enrollment, by intervention group (mITT cohort). (PDF)

S2 Table CONSORT 2010 checklist of information for a randomized trial. (PDF)

S1 Fig Distribution of reported infectious disease symptoms over the first year of life, by baby and intervention group (winter 2011- summer 2015). Each row represents one baby, with each square representing one week of follow-up. Dark (orange) squares indicate a week where the baby was reported as sick; light (grey) squares indicate a week where the baby was reported as healthy; empty squares indicate a week where no survey results were returned. The top panel presents babies in the TC group and the bottom panel presents babies in the non-TC group. Darker columns represent winter months and lighter columns represent summer months.
(TIFF)

S2 Fig Proportion of babies reporting infectious disease symptoms by week over the first year (mPP cohort). (TIF)

S1 Protocol Assessment of urinary triclosan levels. (PDF)

S2 Protocol Stanford’s Outcomes Research in Kids (STORK) Protocol. (PDF)

S1 Text Statistical model. (PDF)

S2 Text READ ME dataset information. (PDF)

S1 File Data dictionaries for datasets. (XLSX)

S2 File Analytic cohort dataset. (CSV)

S3 File mITT analysis dataset. (CSV)

S4 File mPP analysis dataset. (CSV)

S5 File Antibiotic prescription by visit dataset. (CSV)
