# Supplementary material for: Triclosan and triclocarban exposure, infectious disease symptoms and antibiotic prescription in infants—A community-based randomized intervention
Source: PLoS One. 2018 Jun 28;13(6):e0199298. doi: 10.1371/journal.pone.0199298 (PMC6023107; doi:10.1371/journal.pone.0199298)
Supplement: S1 Table — (PDF) [file pone.0199298.s001.pdf]

S1 Table: Maternal and household characteristics at enrollment, by intervention group (mITT cohort)

	TC Arm (n=60)	Non-TC Arm (n=73)	Total (n=133)
First-born child	20 (33%)	21 (29%)	41 (31%)
Maternal age at enrollment (years)			
[mean (SD)]	30 (5.6)	31 (6.1)	31 (5.9)
[median (IQR)]	32 (26-35)	31 (28-35)	31 (27-35)
Maternal race/ethnicity			
Hispanic	38 (63%)	43 (59%)	81 (61%)
White non-Hispanic	10 (17%)	12 (16%)	22 (17%)
Black non-Hispanic	4 (7%)	3 (4%)	7 (5%)
Asian	6 (10%)	12 (16%)	18 (14%)
Not specified	2 (3%)	3 (4%)	5 (4%)
Maternal country of birth			
U.S.	21 (35%)	32 (44%)	53 (40%)
Other	39 (65%)	41 (56%)	80 (60%)
Maternal education level			
Less than high school	19 (32%)	21 (29%)	40 (30%)
High school	12 (20%)	13 (18%)	25 (19%)
Some college	20 (33%)	17 (23%)	37 (28%)
Post college	9 (15%)	22 (30%)	31 (23%)
Household size: median (IQR)	4 (3-6)	4 (3-6)	4 (3-6)
Household with minors (age <18 years)	47 (78%)	55 (75%)	102 (77%)
Minors in household at enrollment			
[mean (SD)]	2.1 (1.3)	1.9 (1.1)	2.0 (1.2)
[median (IQR)]	2 (1-3)	2 (1-2)	2 (1-5)

	TC Arm (n=60)	Non-TC Arm (n=73)	Total (n=133)
Household cleanliness score[a] [median (IQR)]	4 (1-5)	4 (1-5)	4 (1-5)
Crowding (people/room) at enrollment			
[mean (SD)]	1.6 (1.0)	1.3 (0.8)	1.4 (0.9)
[median (IQR)]	1.2 (0.8-2.1)	1.2 (0.8-1.5)	1.2 (0.8-1.8)
Use of cleaning products or chemicals at work			
Yes	15 (25%)	18 (25%)	33 (25%)
No	25 (42%)	28 (38%)	53 (40%)
Not applicable	20 (33%)	27 (37%)	47 (35%)
Bathing habits			
Daily	45 (75%)	53 (73%)	98 (74%)
More than once per day	7 (12%)	12 (16%)	19 (14%)
Every other day	8 (13%)	8 (11%)	16 (12%)

mITT: modified intent to treat; IQR: interquartile range; SD; standard deviation; TC: triclosan/triclocarban

[a] Household cleanliness score (0–10), where 0=very clean; assessed by interviewer.
