# Supplementary figures and images for: Triclosan and triclocarban exposure, infectious disease symptoms and antibiotic prescription in infants—A community-based randomized intervention
Source: PLoS One. 2018 Jun 28;13(6):e0199298. doi: 10.1371/journal.pone.0199298 (PMC6023107; doi:10.1371/journal.pone.0199298)

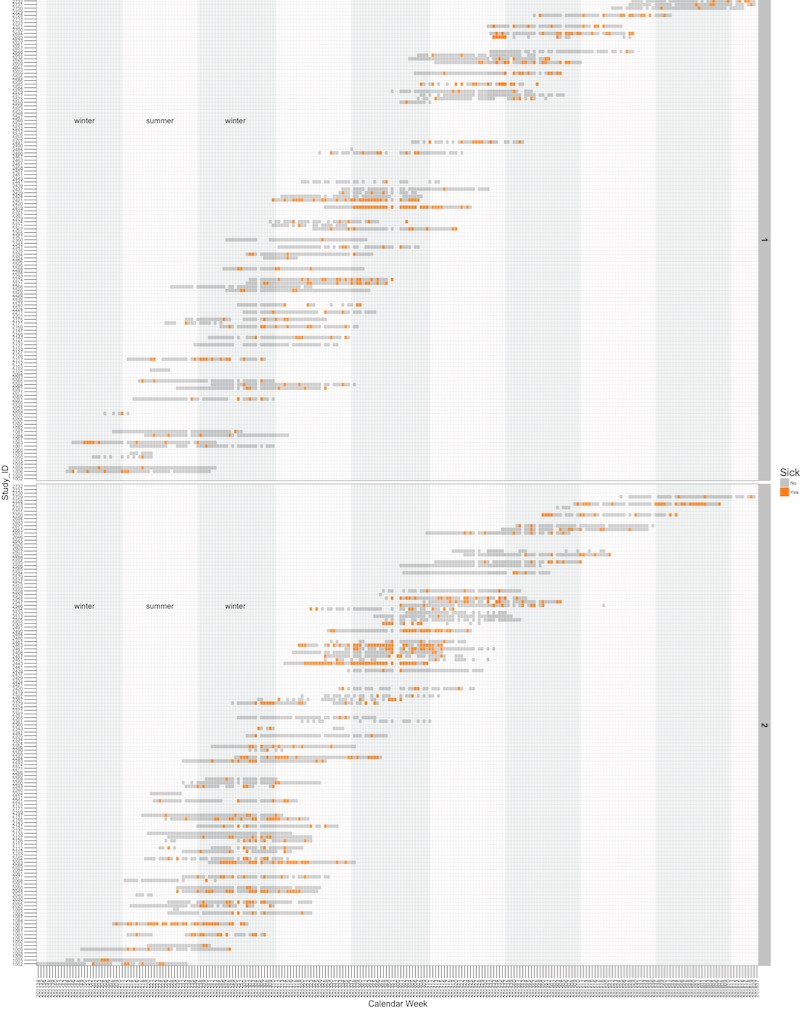

Supplement: S1 Fig — Each row represents one baby, with each square representing one week of follow-up. Dark (orange) squares indicate a week where the baby was reported as sick; light (grey) squares indicate a week where the baby was reported as healthy; empty squares indicate a week where no survey results were returned. The top panel presents babies in the TC group and the bottom panel presents babies in the non-TC group. Darker columns represent winter months and lighter columns represent summer months. (TIFF) [file pone.0199298.s003.tiff]
