# Supplementary material for: Triclosan and triclocarban exposure, infectious disease symptoms and antibiotic prescription in infants—A community-based randomized intervention
Source: PLoS One. 2018 Jun 28;13(6):e0199298. doi: 10.1371/journal.pone.0199298 (PMC6023107; doi:10.1371/journal.pone.0199298)
Supplement: S2 Protocol — (PDF) [file pone.0199298.s006.pdf]

Stanford's Outcomes Research in Kids (STORK)

Protocol, Version 1.2

J Parsonnet , MD
2/26/2014

Modifications

Date	Version	Modifications
14Apr2011	1.1	Removal of all references to the Willow Clinic. This is in order to comply with the Stanford IRB and begin recruitment at the LPCH. (We would need approval from the San Mateo PHD IRB prior to beginning recruitment if the Willow Clinic remained in the protocol.) Sections: Protocol Summary (p. 7); 5.1 (p. 22); 5.2 (p. 23); 17.1 (p. 55)<br><br>Update of Title page, footer; repagination
26Feb2014	1.2	Extension of eligibility age criterion (age 18-42 years) and decrease in exclusion criteria (removal of: history of diagnosis or treatment of type 1 diabetes, major mental illness, thyroid or endocrine condition, severe immunosuppression; household member with compromised immune system)<br><br>Addition of SCVMC Tully Road Clinic<br><br>Modification of compensation for weekly surveys<br><br>Removal of temperature from assessments<br><br>Modification of location where testing for triclosan will be performed.<br><br>Modification of frequency of sampling (stool, urine, saliva, skin swabs) from the mother (every 4 months), and the method of urine collection from the baby.<br><br>Addition of the SCVMC IRB<br><br>Addition of use of electronic data collection via tablet<br><br>Update of Title page, footer; repagination

Statement of compliance

- This study will be conducted in compliance with the protocol, International Conference on Harmonisation of Good Clinical Practice E6 (ICH-GCP) and all applicable regulatory requirements.
- This study complies with provisions of 45 CFR 46 and 21 CFR including parts 50 and 56 concerning informed consent and IRB regulations.
- The study protocol and consent forms have been approved by the sponsoring institution, Stanford University.
- The Principal Investigator has completed human subjects protection training and a course on responsible conduct in research, as required by Stanford University and NIH policies governing clinical awards.

Signature page

The signature below documents the approval of this protocol and the attachments, and provides the necessary assurances that this trial will be conducted according to all stipulations of the protocol, including all statements regarding confidentiality and according to local legal and regulatory requirements and to the principles outlined in applicable US federal regulations and ICH guidelines.

____

Julie Parsonnet, MD – Stanford University

List of abbreviations

AE	Adverse event
BMI	Body mass index (kg/m2)
CDC	Centers for Disease Control and Prevention
CFR	Code of Federal Regulations
CRP	C-reactive protein
DCC	Data Coordinating Center
DNA	Deoxy-ribonucleic acid
DSMB	Data safety and monitoring board
EBV	Epstein Barr virus
ESR	Erythrocyte sedimentation rate
FDA	Food and Drug Administration
HCV	Hepatitis C virus
HIV	Human immunodeficiency virus
ICH-GCP	International conference on harmonisation of good clinical practice
ID	(Personal) identifier
IL	Interleukin
IGF	Insulin-like growth factor
IFN	Interferon
IRB	Institutional Review Board
Kg	Kilogram
LPCH	Lucile Packard Children's Hospital
LPS	Lipopolysaccharide
MIC	Minimum inhibitory concentration
MOS	Margin of safety
MRSA	Methicillin-resistant *Staphylococcus aureus*
NIH	National Institutes of Health
OHRP	Office for Human Research Protections
OTU	Operational taxonomic units
PAI	Plasminogen activator inhibitor
REE	Resting energy expenditure
SAE	Serious adverse event

SCVMC	Santa Clara Valley Medical Center
SMC	Safety monitoring committee
SQL	Structured query language
TNF	Tumor necrosis factor
TBD	To be determined
URI	Upper respiratory infection
US	United States

Protocol summary

**Title**	Stanford's Outcomes Research in Kids (STORK)
**Population**	250 pregnant women attending local public obstetric clinics and subsequently their infants
**Design**	Prospective observational cohort that includes a nested randomized intervention
**Number of sites**	2 (Lucille Packard Children's Hospital, SCVMC Tully Road Clinic)
**Study duration**	9 years
**Subject duration**	3.5 years
**Objectives**	Primary:<br>1. To determine the association between infectious disease load as measured by sick-days from *in utero* to age 3 years, and growth, as measured by height-for-weight Z-score.<br>2. To determine whether exposure to the antimicrobial triclosan, as measured by intervention arm, decreases infectious disease load.<br>Secondary:<br>1. To assess the association between infection and microbial diversity, by characterizing the microbiota by 16S rRNA gene sequence analysis.<br>2. To assess the association between infection and the breadth of the developing immune system response, as measured by the types and proportions of naïve, effector memory and central memory T cells.<br>3. To assess the association between growth and microbial diversity.<br>4. To assess the association between growth and breadth of the developing immune system response.<br>5. To assess the association between triclosan exposure and microbial diversity.<br>6. To assess the association between triclosan exposure and the breadth of the developing immune system response.
**Schematic**	See Figure 1 below

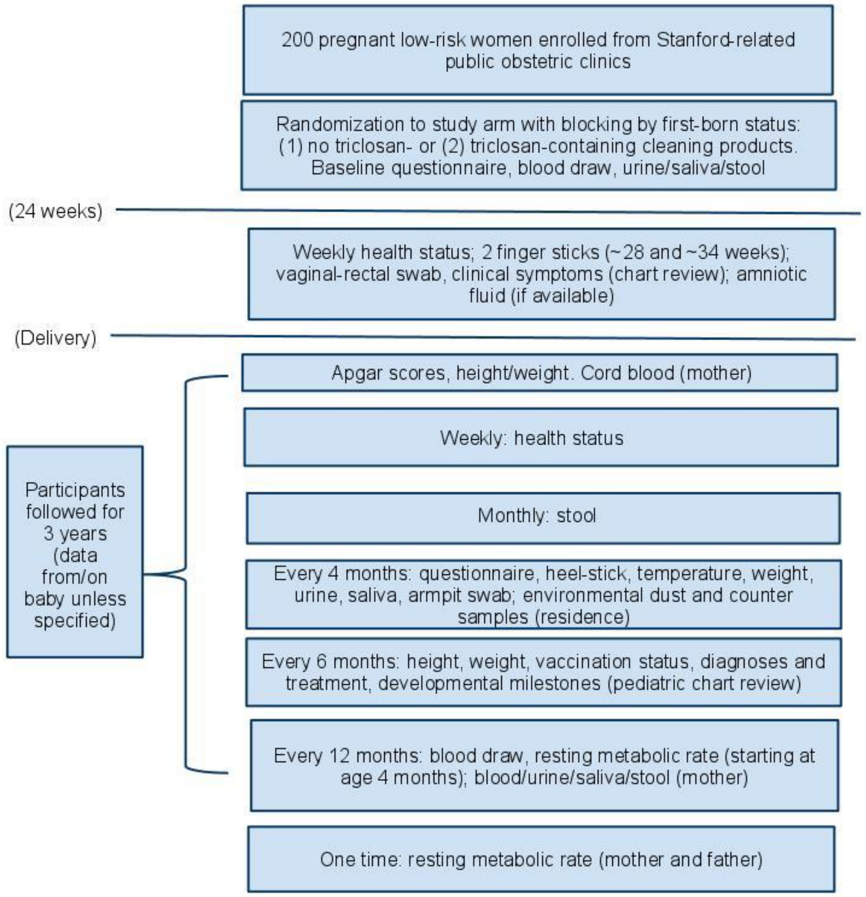

Figure 1: Schematic of STORK

Table of Contents

1. Key roles 13
2. Background information and scientific rationale 13
   2.1. Background information 13
      2.1.1. Description of the study agents(s)/intervention(s) 15
      2.1.2. Summary of previous pre-clinical studies 15
      2.1.3. Summary of relevant clinical studies 16
      2.1.4. Summary of epidemiological data 17
   2.2. Rationale 17
   2.3. Potential risks and benefits 18
      2.3.1. Potential risks 18
      2.3.2. Potential benefits 19
3. Study objectives 20
4. Study design 20
   4.1. Description of the study design 20
   4.2. Study endpoints 21
      4.2.1. Primary endpoint 21
      4.2.2. Secondary endpoints 21
5. Study population 21
   5.1. Description of the study population 21
      5.1.1. Participant inclusion criteria 22
      5.1.2. Participant exclusion criteria 22
   5.2. Strategies for recruitment and retention 23
6. Study agent / interventions 23
   6.1. Study agent acquisition 23
      6.1.1. Formulation, packaging and labeling 24
      6.1.2. Study agent storage and stability 24
      6.1.3. Preparation, administration and dosage of study agent(s)/interventions(s) 24
      6.1.4. Study product accountability procedures 24
   6.2. Assessment of participant compliance with study agent(s)/intervention(s) 24
   6.3. Concomitant medications and procedures 24
   6.4. Precautionary and prohibited medications and procedures 24

6.5. Prophylactic medications and procedures ... 25
6.6. Rescue medications ... 25
7. Study procedures/evaluations ... 25
7.1. Study procedures/evaluations ... 25
7.1.1. Clinical and research laboratory evaluations ... 26
7.1.2. Special assays or procedures ... 27
7.2. Specimen collection, preparation, handling and shipping ... 28
7.2.1. Blood ... 28
7.2.2. Stool ... 28
7.2.3. Urine ... 29
7.2.4. Saliva ... 29
7.2.5. Skin swabs ... 30
7.2.6. Dust swabs ... 30
7.2.7. Kitchen countertop swabs ... 30
8. Study schedule ... 30
8.1. Screening ... 31
8.2. Enrollment/baseline ... 31
8.3. Follow-up ... 31
8.4. Final study visit ... 32
8.5. Early termination visit ... 32
9. Assessment of safety ... 33
9.1. Definition of an adverse event (AE) ... 33
9.2. Definition of a serious adverse event (SAE) ... 33
9.3. Reporting procedures ... 34
9.3.1. Specific serious adverse event requirements ... 34
9.3.2. Reporting of pregnancy ... 34
9.3.3. Procedures to be followed in the event of abnormal laboratory test values or abnormal clinical findings ... 34
9.3.4. Type and duration of the follow-up of participants after adverse events 35
9.4. Halting rules for the protocol ... 35
9.5. Premature withdrawal of a participant ... 35
9.6. Replacement of a participant who discontinues study treatment ... 36
10. Clinical monitoring structure ... 36

10.1. Site monitoring plan 36
10.2. Safety monitoring plan 36
11. Statistical considerations 37
11.1. Overview and study objectives 37
11.2. Analysis plan 40
11.2.1. Cohort 40
11.2.2. Intervention 41
11.3. Sample size considerations 41
11.3.1. Cohort 41
11.3.2. Intervention 42
11.4. Maintenance of trial treatment randomization codes 43
12. Quality control and quality assurance 43
13. Ethics / protection of human subjects 44
13.1. Declaration of Helsinki 44
13.2. Participant confidentiality 44
13.3. Institutional review board/medical ethics committee 45
13.3.1. Assent or informed consent process (in case of a minor) 45
13.4. Exclusion of women, minorities and children (special populations) 45
13.5. Informed consent process 45
13.6. Study discontinuation 46
13.7. Future use of stored specimens 46
14. Data handling and record keeping 46
14.1. Data management responsibilities 47
14.2. Data capture methods 48
14.3. Types of data 48
14.4. Source documents and access to source data/documents 49
14.5. Timing/reports 50
14.6. Study records retention 50
14.7. Protocol deviations 50
14.8. Future use of stored specimens 50
15. Publication policy 51
16. Scientific references 51
17. Appendices 54
17.1. Appendix A1: Site Roster 55

17.2. Appendix A2: Study Contacts Roster 56
17.3. Appendix A3: Consent Form (current) 57
17.4. Appendix A4: Test of Comprehension (current) 58
17.5. Appendix A5: Recruitment Forms 59
17.6. Appendix B: Questionnaires (current) 60
17.6.1. Baseline (household visit) 60
17.6.2. Longitudinal, every 4 months (household visit) 60
17.6.3. Longitudinal, weekly (telephone or email) 60
17.6.4. Chart abstraction 60
17.7. Appendix C: Schedule of Assessments 62
17.8. Appendix D: Manual of Procedures (MOP) 65

1. KEY ROLES

For questions regarding this protocol, contact Catherine Ley at Stanford University School of Medicine, HRP Redwood Building, T225, Stanford CA 94305; cley@stanford.edu.

Principal Investigator:

Julie Parsonnet, MD
Stanford University School of Medicine
300 Pasteur Drive
Grant S125
Stanford, CA 94305-5107
Tel: 650-725-4561
Fax: 650-498-8011
Email: parsonnt@stanford.edu

Institution:

Stanford University School of Medicine
300 Pasteur Drive
Julie Parsonnet, MD
Grant S125
Stanford, CA 94305-5107
Tel: 650-725-4561
Fax: 650-498-8011

See Appendix A1 for the list of study sites and Appendix A2 for the study contacts roster.

2. BACKGROUND INFORMATION AND SCIENTIFIC RATIONALE

2.1. Background information

Obesity and overweight are epidemic in US children. According to the Centers for Disease Control and Prevention (CDC), between the 1960s and today, obesity prevalence more than quadrupled from 4% to 17% (1). Among obese teenagers, 80% will become obese adults, potentially suffering the gamut of weight-related problems. Physiologically, only imbalance between caloric intake and expenditure can explain the shifts in weight. However, diet and exercise--the primary recipients of research attention with respect to intake and expenditure--do not correlate with childhood obesity as much as might be expected (2). Moreover, randomized clinical trials addressing diet and exercise have demonstrated little to no impact on childhood body mass index (BMI) indicating that, even if they are important, they are difficult to modify (3). We hypothesize that the rise in childhood obesity is caused by the loss of recurrent and chronic infections in modern, industrialized society, beginning in *utero* and extending through early childhood.

Surprisingly little is known about the sequence or frequency of infection in childhood or infections' metabolic toll. In the extreme, however, few would disagree that infection protects against obesity. Children who suffer severe, recurrent infection - e.g. children in the developing world - are rarely, if ever, fat. In the industrialized world, recurrent infections in cystic fibrosis result in heightened resting energy expenditure (REE) and poor weight gain despite good caloric intake (4). Although these extremes may have little relevance to average children, the hypothesis that infection decreases weight in normal children is certainly plausible. Infections both increase expenditure and decrease caloric intake. Each degree Celsius elevation in temperature results in 7-16% increase in REE (5, 6).

Fever is not the only consideration in increased energy expenditure. Despite similar temperature curves and caloric intake during their period of illness, infants with neonatal sepsis have 120% the REE with 35% the weight gain of non-septic neonates (7). Of course, just because temperature and REE are acutely elevated during fever doesn't mean that children will lose weight over a longer term. In fact, some studies indicate a rebound in weight after some acute illnesses (5). However, chronic infections - e.g. asymptomatic HIV, bacteriuria, and schistosomiasis - have all been associated with prolonged REE elevations (8-10), as has elevation in C-reactive protein (11). Similarly, treatment of hepatitis C virus (HCV) results in reduction of REE (12). Adipocytokines, too, may be affected by infection. For example, eradication of *Helicobacter pylori* in pre-pubertal children decreases ghrelin levels and increases BMI compared to untreated children (13). In fact, most chronic infections - symptomatic and asymptomatic - are likely to increase metabolic demands in their constant cross-talk with the host. Other mechanisms may be in play as well. Inflammatory cytokines suppress appetite and affect cell differentiation. Specifically, circulating tumor necrosis factor (TNF) both inhibits adipocyte differentiation and promotes lipolysis (14). In this manner, short-term infections can have long-term effects on very young children.

Historically, the greatest shifts in population health have been attributable not to changes in genetics or human behavior, but to alterations in infectious disease incidence. Due to decreasing family size (also linked to obesity), improvements in hygiene, expanded spectrum of vaccine coverage, and early antimicrobial use, infectious diseases are simply disappearing as a serious problem in children of the US and the industrialized West (15, 16). Studies document declining incidence of rheumatic fever, scarlet fever (17), post-streptococcocal glomerulonephritis (18), tuberculosis, Epstein Barr virus (EBV), toxoplasmosis, invasive group B streptococcal infections (19), childhood diarrhea (20), appendicitis (21), erysiplelas, otitis media (22), dysentery, *H. pylori*, and vaccine preventable diseases. Furthermore, as infection incidence diminishes, the age of infection acquisition rises, potentially affecting cell growth and differentiation. What overall consequences is this ecologic shift going to have?

Changes are likely occurring also in the microbiota of the human gastrointestinal tract, which can itself be considered infectious – i.e., acquired from the environment. Gut microbial populations have been shown to impact a variety of metabolic processes including inflammation, angiogenesis, immunity, and insulin resistance (23). They may have an important role in growth, as relative proportions of bacterial phyla have been shown to be responsive to weight change (24). Thus, childhood infectious conditions,

some of which dramatically impact the gut microbiota at least in the short term, may well have long-term effects on the microbiota and as such may well play a role in long-term weight change.

The hypothesis that childhood infections protect against obesity is revolutionary and potentially paradigm shifting. If true, it raises a critical question: is increasing weight in childhood a sign of good health or of disease? No one would argue that morbid obesity in adulthood is healthy. Increased weight in children, however, is a poor predictor of adult obesity. Moreover, even countries in socioeconomic transition like China, India and Mexico are seeing increases in childhood weight, particularly among the upper classes - an observation that is likely to mark improved longevity. The discovery that overweight in childhood results from our own remarkable progress in protecting children from premature death from infection may, in the end, transform our concepts of obesity and lead us to consider more carefully how we modulate our relationship with the microbial world.

2.1.1. Description of the study agents(s)/intervention(s)

Triclosan (5-chloro-2-(2,4-dichlorophenoxy)-phenol) is a chlorinated phenolic antibacterial compound found in consumer products since 1972, including deodorants, toothpastes, shaving cream, mouth wash, cleaning supplies, kitchen utensils, toys, bedding, socks, and trash bags (25). Triclosan is regulated by the FDA, which is currently conducting a review on its safety (26). To date, triclosan has been proven to reduce plague and gingivitis when used in toothpaste; no other evidence showing an extra benefit to health for triclosan in other consumer products has been demonstrated. The mechanism of action at higher concentrations is non-specific but at lower concentrations, appears to specifically target bacterial fatty acid synthesis. These antibiotic properties have led to concerns about development of antimicrobial resistance. In addition, there is evidence that triclosan may be an endocrine disruptor in animal models. Triclosan has been found in serum, urine, breast milk, sewage and in drinking water in small amounts and has been banned in Denmark, Norway, Sweden, the United Kingdom, and Finland.

While probably reducing microbial diversity in the home environment, triclosan may have subtle adverse health effects that remain to be identified. Within the cohort study presented below, we are nesting an intervention: the randomized removal of triclosan-containing cleaning products from the household. Participants will be randomly assigned to receive household cleaning products either of their choice or not containing triclosan, with the products supplied on an as-needed basis. Because the purchase of anti-microbial containing products is done presumably to reduce the incidence of infection within the household, we will assess the incidence of infectious disease in each infant and compare the rates in both arms.

2.1.2. Summary of previous pre-clinical studies

Triclosan has been used extensively for more than 40 years. It is an off-white odorless tastelss crystalline powder, practically insoluble in water, moderately soluble in alkaline solutions and readily soluble in most non-polar organic solvents (27).

Triclosan's activity is concentration and formulation dependent. The primary site of its action is the cytoplasmic membrane, with the uptake of triclosan by the cell wall, as well as whole cells being by diffusion. The primary antimicrobial action of triclosan is directed toward RNA and protein synthesis in bacteria, and not against DNA. It has been shown to be effective in low concentrations against a broad spectrum of gram-negative and gram-positive bacteria, notably Proteus vulgaris, Salmonella sp., mycobacteria, anaerobic bacteria, and fungi, ammonia producing bacteria, yeast, dermatophytes, and certain resident skin bacteria.

Triclosan is not acutely toxic to mammals as shown by traditional toxicity tests in mice, hogs or rats (27). Subacute/subchronic toxicity tests in rats, rabbits, dogs and baboons ranging from 3 days to 52 weeks assessed the potential effects of oral and dermal applications. Toxic effects differed by species and route of administration: hepatic lesions and hematologic effects occurred with oral administration by intubation or capsule in rabbits and dogs; baboons showed only episodes of emesis and diarrhea.

Chronic toxicity / carcinogenicity studies did not identify a carcinogenic potential at any treatment level in rats (27). Treatment-related tumors were found only in the liver of male and female mice; these tumors were suggested to have arisen by way of peroxisome proliferator-activated receptor alpha (PPARalpha) activation, a mode of action not considered to be relevant to humans (28).

Numerous independent mutagenicity studies did not identify positive results (27). A reproductive study in rats showed no effect on reproductive performance and in studies conducted to assess developmental toxicity in rats and rabbits, teratologic effects were not observed.

Triclosan has been shown in vitro to activate the human pregnane X receptor (29), to induce hepatic phase I enzymes and in vivo to cause a decrease in total thyroxine (T4) (30, 31), suggesting adverse effects on thyroid hormone homeostasis. However, the data appear to be of questionable physiological relevance, as there is no evidence of hypothyroid effects or adjustment of the hypothalamo-pituitary axis to this decreased T4.

Triclosan exhibits relatively high affinity for the androgen receptor: in cell-based bioassays, it exhibits androgen antagonist activity without any agonist activity (32). This antiandrogenic effect is not demonstrable in vivo.

2.1.3. Summary of relevant clinical studies

The tolerability and safety of triclosan has been evaluated in human volunteers with little indication of toxicity or sensitization (28). Information in humans from chronic usage of personal care products is not available. A Benchmark Dose (BMDL(10)) of 47 mg/kg/day was developed based on kidney toxicity in the hamster. Estimates of the amount of intake from in the use of representative personal care products for men, women, and children were derived in two ways: (a) using known or assumed triclosan levels in various consumer products and assumed usage patterns (product-based estimates); and (b) using upper bound measured urinary triclosan levels from human volunteers (biomonitoring-based estimates) using data from the Centers for Disease Control and Prevention. For the product-based estimates, the margin of safety (MOS) for

the combined exposure estimates of intake from the use of all triclosan-containing products considered were approximately 1000, 730, and 630 for men, women, and children, respectively. The MOS calculated from the biomonitoring-based estimated intakes were 5200, 6700, and 11,750 for men, women, and children, respectively. Based on these results, exposure to triclosan in consumer products was not expected to cause adverse health effects in children or adults who used these products as intended.

Triclosan is being or has been tested in a total of 26 trials in oral health, three trials in MRSA, and 13 trials related to in surgery, wound or stents listed on ClinicalTrials.gov (33). To date, data on the effect of triclosan in oral health outcomes are clear. Triclosan ⁄ copolymer dentifrices containing 0.3% triclosan, 2.0% copolymer and fluoride (1100 ⁄ 1450 p.p.m. F) (Colgate Total, Colgate-Palmolive company, NJ, USA) maintain concentrations of triclosan in plaque that exceed the minimum inhibitory concentration (MIC) values of many plaque bacteria for up to 12 h. Numerous randomized, controlled clinical trials and reviews have shown significant efficacy of this triclosan ⁄ copolymer dentifrice in reducing plaque and improving gingival health and peridonatal disease. Triclosan ⁄ zinc citrate dentifrices have also shown excellent efficacy in gingival health.

With respect to MRSA, one observational report found that the addition of a 0.3% triclosan soap was instrumental in containing an outbreak of MRSA in a neonatal nursing unit and preventing recurrence for 3½ years. A study from the UK suggested that widespread commercial use was not significantly increasing rates of resistance (34). Triclosan has been considered as a potentially useful adjuvant to topical agents, but optimal treatment plans for aggressive decolonization of MRSA are as yet undefined.

2.1.4. Summary of epidemiological data

A systematic literature review assessed 27 studies published between 1980 and 2006 that had examined the efficacy of products containing triclosan, compared with that of plain soap, in the community setting, as well as to evaluate emergence of antibiotic-resistant bacteria (35). Soaps containing triclosan within the range of concentrations commonly used in the community setting (0.1%–0.45% wt/vol) were no more effective than plain soap at preventing infectious illness symptoms and reducing bacterial levels on the hands. Several laboratory studies demonstrated evidence of triclosan-adapted cross-resistance to antibiotics among different species of bacteria. The authors suggested that the lack of an additional health benefit associated with the use of triclosan-containing consumer soaps over regular soap, coupled with laboratory data demonstrating a potential risk of selecting for drug resistance, warranted further evaluation by governmental regulators regarding antibacterial product claims and advertising.

2.2. Rationale

Global infectious disease burden is decreasing dramatically, with standards of living increasing and improvement in medical care access around the world. Concurrently, the global population is getting fatter and fatter. Diet and exercise alone cannot explain this rise in obesity; is it partially attributable to the absence of infection? This study will

examine whether decreased infectious disease load is associated with increased growth as measured by height-for-weight.

Additionally, data on how both growth and infectious disease load affect the diversity of the microbiome in people are few. While details on how growth or infectious disease load affect the immune system response in very young children are less sparse, a greater understanding of these relationships would be helpful. This study will provide information on how these factors relate to each other.

Numerous antibacterial products are widely commercially available, presumably having the ultimate goal of reducing infection in those who use them; however, there is no evidence that these products have a measurable effect on infectious disease incidence. Additionally, the impact that these products may have on the gut microbiome is unknown. This study will examine whether exposure to the antimicrobial triclosan is associated with a decreased incidence of infection within a household. It will also examine whether triclosan exposure is associated with decreased microbial diversity in people, or decreased breadth of immune system response.

Our specific study cohort will be comprised of 200 babies born from low risk, primarily low income women locally resident in the San Francisco Bay area, mostly of Hispanic or recent immigrant origin. Low income families suffer disproportionately from infections due to their increased likelihood of crowding; women in these households also are more likely to be exposed to triclosan through their occupation as house-cleaners and thus may be particularly interested in the study hypothesis. As such, this population could benefit from the added information that our study will make accessible to them. Furthermore, the relationships examined in this study sample represent those in billions of individuals worldwide.

2.3. Potential risks and benefits

2.3.1. Potential risks

<u>1. Immediate risks</u>

A total of 250 pregnant women will undergo one blood draw with venipuncture and up to two finger-sticks during their pregnancy, if not already part of their prenatal care, and up to 200 infants will undergo both an annual blood draw for a total of three each and every-four-month heel sticks for a total of six each. There is a small risk of bruising with venipuncture, for which precautions are taken. Infection at the site of phlebotomy is quite rare; all efforts will be made to prevent this complication by use of trained phlebotomists with great attention to sterile technique. Fainting can occur with phlebotomy in adults. Such risk will be minimized by identifying patients who have fainted previously and requiring them to lie down for phlebotomy. Others without such a history will have blood drawn in a sitting position and will be monitored for complaints or signs of lightheadedness. Blood draw volumes are within guidelines for healthy adults and age-appropriate for infants.

The women may be considered to be from vulnerable populations, due to language barriers as well as factors related to economic, social resettlement and potentially anxiety

about immigration status. Study personnel will be made cognizant of the anxieties attendant to immigrants and will not put undue pressure on participants or potential participants. Precautions will be taken as part of the consent process to insure communication facility as well as understanding that health needs and benefits will not be affected by participation or nonparticipation in the study (see Appendix A3, Consent form). We will require that subjects be able to communicate effectively in Spanish or English. All forms will be translated into Spanish for Spanish speaking subjects. Tests of comprehension in Spanish or in English will be administered at the end of each informed consent process to ensure understanding (see Appendix A4, Test of comprehension).

It is unclear whether triclosan has negative health effects. It is clear that removal of triclosan from products other than toothpaste has no health risk. It is unknown whether continued use of triclosan-containing products already present in the home environment will have any risk to the health of the family.

<u>2. Long range risks</u>

There are no long health term risks, other than prolonged treatment for short-term adverse effects.

There is a small confidentiality risk in the use of mobile phones to contact participants on a weekly basis.

<u>3. Rationale for the necessity of such risks</u>

Weekly health status reports are an essential aspect to this study. The security risk of having a participant identified by their mobile phone number is low.

<u>4. Alternative data gathering procedures that have been considered or will be considered</u>

Participants will be given the option of using email as an alternative for providing weekly updates.

<u>5. Why alternative procedures may not be feasible</u>

The sample will be comprised of primarily immigrant women whose lower socio-economic status, lower literacy rates and low income in general may not allow them regular access to the internet for email updates on health status.

<u>6. Why the value of the information to be gained outweighs the risks involved</u>

Women with small children often have mobile phones and in focus group discussions about the study have indicated a willingness to respond to a weekly telephone survey.

2.3.2. Potential benefits

Because it is unclear whether triclosan has negative health benefits in humans, it is unknown whether removal of triclosan in the home environment will have any benefit on the health of the family.

Because triclosan-containing products tend to cost more than products that do not contain triclosan, a clear understanding of whether the increased cost of these products has any associated benefit would be of assistance to families concerned with their budget.

From a societal perspective, better knowledge of how infection and host immune responses affect body habitus may lead to more effective prevention and treatment strategies.

3. STUDY OBJECTIVES

Our objectives are:

1. A. To determine the association between infectious disease load from in *utero* to age 3 years, as measured by sick-days, and growth, as measured by height-for-weight Z-score.

1. B To determine whether exposure to the antimicrobial triclosan, as measured by intervention arm, decreases infectious disease load, as measured above.

2. A. Within the cohort study:

- - To assess the association between infection and microbial diversity, by characterizing the microbiota by 16S rRNA gene sequence analysis.
- To assess the association between infection and the breadth of the developing immune system response, as measured by the types and proportions of naïve, effector memory and central memory T cells.
- To assess the association between growth and microbial diversity.
- To assess the association between growth and breadth of the developing immune system response.

2. B. Within the intervention:

- To assess the association between triclosan exposure and microbial diversity.
- To assess the association between triclosan exposure and the breadth of the developing immune system response.

4. STUDY DESIGN

4.1. Description of the study design

This observational cohort study recruits pregnant women to follow their infants in *utero* and after birth for three years with the goal of assessing infectious disease burden and changes in growth, resting metabolic rate, immune system development and microbiome development. Mothers will report any infectious conditions during pregnancy and then report the health status of their baby on a weekly basis after delivery; every six months, height and weight measurements, infectious disease diagnoses and treatments, and developmental milestones will be obtained from the pediatric record; every four months, temperature, weight, a saliva sample, a skin swab and environmental samples will be taken and blood drawn to assess the cumulative presence of antibodies to infectious

agents; annually, markers of immune system development will be examined and resting metabolic rate will be measured. Additionally, the diversity of the microbiome will be assessed over time from monthly stool samples.

Superimposed on this cohort study is a randomized intervention of the removal of triclosan-containing cleaning products from household. The goal will be to correlate the presence or absence of triclosan with infectious disease burden and microbiome diversity. Because first-born children are both at decreased risk of infection and at greater risk of increased weight gain, the randomization will be stratified by first-born status, to generate an equal proportion of mothers with first-born children in each trial arm. Household and personal cleaning products will be provided to each participant every four months; the urinary concentration of triclosan will be assessed every four months in infants and annually in mothers.

4.2. Study endpoints

4.2.1. Primary endpoint

The primary outcome for the cohort study is growth as measured by height-for-weight Z-score. We will determine whether an association exists between height-for-weight Z-score (with adjustment for age and sex) and infectious disease load.

The primary outcome for the intervention is incidence of infection as measured by cumulative sick-days from *in utero* to age three years. We will determine whether an association exists between intervention arm and sick-days, adjusted for disease severity.

4.2.2. Secondary endpoints

Key secondary endpoints include:

- Microbial diversity, by its characterization by 16S rRNA gene sequence analysis.
- The breadth of the developing immune system response, as measured by the type and proportions of naïve, effector memory and central memory T cells.

5. STUDY POPULATION

5.1. Description of the study population

The study sample will be enrolled from the population of pregnant women attending Stanford-related obstetric clinics for the first antenatal visit. Each woman will be followed throughout her pregnancy and then she and her baby will be followed over the first three years of the baby's life (and more if possible).

We seek to enroll women who are primarily of lower SES and of Hispanic or immigrant origin. Infectious conditions are known to be more common in families where crowding is high. Families with lower SES tend to live in more crowded conditions; additionally,

Hispanic and immigrant families are more likely to incur infections more prevalent in their country of origin (e.g., *H. pylori*). Furthermore, in the local area, both lower SES and Hispanic ethnicity are associated with increased risk of obesity in adults.

It is possible that oversampling in this community may lead to insufficient variation in the incidence of infection, with all families incurring a high load of infection. It is also possible that, due to the high background rate of obesity in this community, the sample may result in a high proportion of infants with an elevated BMI for sex and age.

5.1.1. Participant inclusion criteria

All subjects will undergo an assessment in which inclusion and exclusion criteria are reviewed by study personnel prior to inclusion in the cohort.

Subjects must meet all of the following inclusion criteria to participate in the study:

- Able to provide written informed consent.
- Able to communicate effectively in verbal English or Spanish. A test of comprehension of the study is included for all subjects as part of the consent process.
- Female adult (18-42 years of age) within the first two trimesters (24 weeks) of pregnancy.
- Willing to provide blood, saliva, urine and stool specimens from herself, and willing to provide blood, saliva, urine and stool specimens from her infant.
- Willing to provide consent for review of Primary Provider Medical Records regarding, for the mother, medical history and infectious and other conditions during pregnancy, and for the infant, Apgar score, height and weight measurements at birth and over time, infectious and other disease diagnoses and treatment, and vaccination records.

5.1.2. Participant exclusion criteria

Subjects meeting any of the following exclusion criteria will be excluded from study participation:

- More than one fetus.
- High risk pregnancy.
- Intent to move from the Bay Area within three years after enrollment.
- Other conditions that in the opinion of the PI would make the subject an unacceptable candidate for this study.

5.1.2.1. Co-enrollment guidelines

A study participant many be concurrently enrolled in another research study or may choose to enroll in another study while participating in this study, at the discretion and with the approval of the PI.

5.1.2.2. Termination criteria

Women who miscarry their fetus will be considered terminated from the study and will be replaced.

Children who die or who are removed from their family for any reason within the first six months of life will be replaced. Those who die or are removed within the subsequent two and one half years of follow-up will be considered terminated from the study and will not be replaced.

5.2. Strategies for recruitment and retention

The study population will be drawn from the communities of East Palo Alto / East Menlo Park in San Mateo County and of San Jose in Santa Clara County and recruited through participation in public obstetric clinics at a first or subsequent prenatal visit. The obstetrics clinics that have agreed to participate to date are the Lucile Packard Children's Hospital (LPCH) (Stanford, CA) and the Santa Clara Valley Medical Center (SCVMC) Tully Road Clinic (San Jose, CA). These clinics provide a referral pool enriched toward our enrollment targets, including a high proportion of Hispanic and foreign-born women from countries where infections are endemic.

Women interested in the study based on a query from her clinician or the clinic staff will be contacted by Stanford personnel, screened for eligibility and invited to participate. Enrolled subjects will be provided free household and personal cleaning products every 4 months as part of the intervention. Compensation for participation will include at every visit a $20 gift card to one of several local stores (Safeway, Target and/or Walmart). Given that this study, as approved by the Stanford IRB, is considered inherently ethical with low risk and a correspondingly good positive benefit/risk ratio, this small amount of money is not considered coercive or undue inducement, even for an immigrant group, provided informed consent is appropriately obtained. An alternative incentive may include a voucher for the local Farmer's Market. Participants will receive a $25 gift card for every 24 weekly surveys completed. A series of presentations by clinicians at the local clinics is planned, with short talks on topics of interest to the study sample and the clinic population, such as an overview of infectious diseases in childhood.

A draft recruitment form can be found in Appendix A5.

6. STUDY AGENT / INTERVENTIONS

6.1. Study agent acquisition

Household and personal cleaning products available at high volume commercial retailers will be delivered to the home. Two lists, one with all items and one with only those items that do not contain triclosan, will be generated from the available merchandise in the following categories:

- Household cleaning products (hand dishwashing detergents)

- Personal care products (hand soaps, bar soaps, toothpaste)

Participants will be provided with the appropriate list (non-triclosan containing products only or all) and will select those items to be sent to the household on a quarterly basis or as needed.

Categories will be reviewed on a regular basis for any new items, so that study staff are aware whether or not they contain triclosan.

6.1.1. Formulation, packaging and labeling

Products containing triclosan acquired for this study will be formulated, packaged and labeled by the original manufacturer with no modification for this study.

6.1.2. Study agent storage and stability

Products containing triclosan acquired for this study will be obtained from a high volume commercial retailer (e.g., Safeway) which maintains a high-quality supply chain. If the participant decides that the product does not meet reasonable standards of stability and storage, then the product will be replaced.

6.1.3. Preparation, administration and dosage of study agent(s)/interventions(s)

Products containing triclosan will be provided to participants if they choose to select these products. Use of these products will be as per each individual product's label and at the discretion of the participant.

6.1.4. Study product accountability procedures

Products will be provided to the participant on the assumption that the products will be used within the household by household members only. On the assumption of reasonable use, the products will be replaced quarterly or as needed. In the case of excessive amounts of products being used by the household, the study coordinator will assess whether this use is justified.

6.2. Assessment of participant compliance with study agent(s)/intervention(s)

Participants are free to use or not use the products provided in the study.

6.3. Concomitant medications and procedures

No concomitant medications or procedures beyond the randomization to triclosan or non-triclosan containing cleaning products are used in this study.

6.4. Precautionary and prohibited medications and procedures

Because the one known health benefit of triclosan is its positive effect on gingivitis and peridontal disease, we will not require households randomized to the non-triclosan arm to

remove any triclosan-containing toothpaste from the home. No medications or procedures are considered to be precautionary or prohibited.

6.5. Prophylactic medications and procedures

Because triclosan is not administered or dosed in this study, but is simply present in each participant's individual environment at work and at home in unknown amounts, no adverse events due to triclosan exposure will be identified. No prophylactic medications or procedures are required.

6.6. Rescue medications

Because triclosan is not administered or dosed in this study, but is simply present in each participant's individual environment at work and at home in unknown amounts, no adverse events due to triclosan exposure will be identified. No rescue medications are required.

7. STUDY PROCEDURES/EVALUATIONS

7.1. Study procedures/evaluations

Information will be collected and recorded by trained study personnel using standardized, pre- tested forms that are either paper or electronic (see Appendix C, Questionnaires). [For details on the sequence of procedures and evaluations, see Section 8: Study Schedule, and Appendix D, Schedule of Assessments.]

<u>1. Evaluations</u>

Evaluations in this study are either interviewer-administered questionnaires, self-reported responses via a telephone or email survey, chart abstraction, laboratory findings from biological and environmental specimens, or measurements from temperature, weight and resting metabolic rate assessments.

- Questionnaires. Questionnaires cover the following information:
    - Patient demographics, family structure (household size, sibship structure, birth order, country of origin, time in the US, level of education obtained), participation in out-of-home child care, measures of crowding, assessment of household cleanliness.
    - Weekly report of health status during pregnancy for the mother and of the infant's health status after delivery.
- Chart abstraction. Primary Provider Medical Records abstraction includes data on: (1) for the mother, during pregnancy: pre-pregnancy weight and weight gain over time, reproductive history, chronic conditions and treatments, infectious disease diagnoses and treatments, and other diagnoses and treatments; (2) for the baby, at delivery: Apgar scores, height, weight, complications at birth; at other visits

(well-baby or unscheduled): height and weight, vaccination history, infectious disease diagnoses and treatments, other diagnoses and treatments, and developmental milestones.

- Specimens. Specimens will be collected from both the mother and the infant including: from the mother: blood, stool, saliva, arm-fold skin swab and urine, amniotic fluid if available and cord blood at delivery; from the child: blood, stool, saliva, urine, an armpit skin swab. Samples will also be collected from under the baby's sleeping place, and from the kitchen counter.
- Weight and resting metabolic rate

<u>2. Procedures</u>

Procedures used in this study include:

- Blood draw. Phlebotomy will be used to collect blood samples from both the mother (at baseline) and the infant (annually). Finger pricks will be used to collect samples from the mother (up to twice prior to delivery). Heel sticks will be used to collect samples from the infant (every four months except at the annual blood draw).
- Stool collection. Mothers will be provided kits with instructions for collecting stool, and how to then store these samples and have them be transported to the laboratory for processing.
- Indirect calorimetry. Indirect calorimetry will be used as a measure of resting metabolic rate, measured once in the mother (after delivery) and the father and three times in the infant (at 4, 16 and 28 months) (see Appendix D: MOP).
- Weight. Weight of the infant will be taken every four months using a portable scale (see Appendix D: MOP).
- Saliva collection. Saliva will be collected every four months from the baby and from the mother.
- Armpit swab. A skin swab from under the armpit will be collected every four months from the baby and from the arm-fold from the mother.
- Laboratory evaluations/assays

7.1.1. Clinical and research laboratory evaluations

Table 1 below outlines the specimens to be collected and laboratory evaluations to be conducted. Reference ranges will be included in the MOP (see Appendix D).

Table 1: Specimens to be collected and laboratory evaluations to be conducted

Specimen type	Volume	Specification	Assessment
**MOTHER**			
Blood	2 X 0.3 ml	Gold/red top tube	Antibodies

	3 X 5 ml	Gold red top tube	TNF, grehlin, insulin, leptin, IFN-gamma, IGF, glucagon, resistin, PAI-1, IL10, IL17
	3 X 2 ml	Purple top tube	CRP, ESR
Stool	4 X	Swab, container	Microbiome
Saliva	4 X	Swab, tube	Microbiome
Urine	4 X 10 ml	Container	Triclosan
Vaginal-rectal swab	1 X	Swab, container	Microbiome
Amniotic fluid	1 X 0.3 ml	Small tube	Antibodies
Cord blood	1 X 0.3?	Small tube	Antibodies
Dust	9 X	Swab, container	Triclosan
Countertop swab	9 X	Swab, container	Microbiome
**CHILD**			
Blood	6 X 0.3	Gold/red top tube	Antibodies
	3 X 3 ml	Gold/red top tube	TNF, grehlin, insulin, leptin, IFN-gamma, IGF, glucagon, resistin, plasminogen activator inhibitor 1 (*PAI-1*), IL10, IL17
	3 X 0.5	Purple top tube	CRP, ESR
	3 X 0.9 ml	TBD	LPS (lipopolysaccharide, a marker of inflammation due to bacteria)
Stool	36 X	Swab, container	Microbiome
Saliva	9 X	Swab, container	Microbiome
Urine	9 X 3 ml	Container	Triclosan
Skin swab	9 X	Swab, tube 1 ml 0.15 M NaCL and 0.1% Tween 20	Microbiome

7.1.2. Special assays or procedures

The following assays will be done at the Parsonnet lab within 6 hours of drawing the sample:

- CRP
- ESR

Details of these assays will not be discussed further here.

Other assays will be done in research laboratories. These include:

- Adipocytokines, interferon gamma, leptin, ghrelin, glucagon. Samples will be batched and shipped on dry ice to Eve Technologies. Custom Luminex kits will be run on serum.
- Triclosan. Testing of urine samples will be performed at Stanford. Samples will be batched and stored at or below -20°C until analyzed. Testing of environmental samples (dust swabs) will be performed by the Woods Institute at Stanford.
- Stool, saliva, vaginal-rectal swab. PCR-product will be batched and shipped on dry ice to the Environmental Genomics Core facility (EnGenCore) at the University of South Carolina or other appropriate facility and stored at or below -20°C until analyzed.
- LPS - TBD
- Antibodies - TBD

7.2. Specimen collection, preparation, handling and shipping

7.2.1. Blood

Blood samples will be collected by a qualified phlebotomist according to standard procedures and precautions. The blood draw will take place at the end of the interview. Samples will be labeled with a unique ID and the date of collection. Tubes will be placed in a cooler to transport them to the Parsonnet Lab.

Blood samples will be logged at the Parsonnet Lab by unique ID, date of collection and initials of the person receiving them.

Tubes will be spun to 3,000 g x 10 minutes to separate plasma or serum. Serum or plasma will be aliquoted in 200 ul and stored at -80°C for future testing.

7.2.2. Stool

Stool samples will be collected in an empty wide mouth (50 ml) container, up to monthly from the baby by the mother, and every 4 months from the mother.

Mother. Participants will have received a kit for stool collection. The kit will include one container pre labeled with unique ID and collection date, one paper hat, tongue suppressor and instructions for sample collection and mailing.

Baby. Participants will have received a kit for stool collection and mailing. The sample will be collected from a diaper soiled the same day. Using a tongue suppressor or swab,

10-25 g of stool will be transferred from the diaper to a wide mouth container, pre labeled with unique ID and collection date.

For months where the STORK study team is visiting the participant, a diaper can be retained and stool collected by the study team. The sample will be placed in a cooler to transport it to the Parsonnet Lab.

Stool samples will be logged at the Parsonnet Lab by unique ID, date of collection and initials of the person receiving them.

Received stool samples will be transferred to a 0.5 ml cryovial to be stored at -80°C for future use.

To generate PCR product, the frozen stool samples will be ground under liquid N_2, suspended while frozen in a solution containing DNA extraction buffer, 20% SDS, a mixture of phenol:chloroform:isoamyl alcohol and a slurry of 0.1-mm-diameter zirconia/silica beads. Microbial cells will then be lysed by mechanical disruption with a bead beater, followed by extraction with phenol:chloroform:isoamyl alcohol, and precipitation with isopropanol. The quantity and quality of purified DNA will be assessed. 16S rRNA genes will then be amplified from each sample. PCR product will be stored at -80°C until shipped for future analysis as above.

7.2.3. Urine

Participants will receive a urine collection kit that will include: a container or a urine collection bag (pediatric) and instructions for collection. Samples will be labeled with the unique ID and date of collection.

Mother. Participants will have received a kit for urine collection. Samples will be pre-labeled with the unique ID and date of collection.

Baby. The sample will be collected using a pediatric urine collection kit the same day. Urine will be transferred to a container, pre labeled with unique ID and collection date.

Samples will be placed in a cooler to transport them to the Parsonnet Lab.

Urine samples will be logged at the Parsonnet Lab by unique ID, date of collection and initials of the person receiving them.

A minimum of 5 ml of the urine will be transferred to specimen vials within 4 hours of collection to a poly propylene vial. Crimped caps with rubber stoppers will not be used because of their composition. The specimens will then be labeled and frozen immediately to -20 °C.

7.2.4. Saliva

We will ask the participant to place a sterile swab in her mouth for a short time until the swab becomes soaked in saliva. This process will be repeated if the swab is not well soaked in saliva. In the same way, a swab will be placed in the baby's mouth. The swab will be placed in a tube with storage solution. Tubes will be labeled with the unique ID and date of collection.

The tubes will be placed in a cooler for transportation to the Parsonnet Lab.

Swab samples will be logged at the Parsonnet Lab by unique ID, date of collection and initials of the person receiving them.

Swab samples will be stored at -80° for future use. PCR product will be generated as above.

7.2.5. Skin swabs

For each baby, the armpit will be swabbed with a cotton-tipped swab moistened with a solution of 0.15 M NaCl and 0.1% Tween 20. Tubes will be labeled with the unique ID and date of collection.

The tubes will be placed in a cooler for transportation to the Parsonnet Lab.

Swab samples will be logged at the Parsonnet Lab by unique ID, date of collection and initials of the person receiving them.

Swab samples will be stored at -80° for future use. PCR product will be generated as above.

7.2.6. Dust swabs

At each household, a 10 cm square area under the baby's beds will be swabbed with a cotton tip swab moistened with a solution of 0.15 M NaCl and 0.1% Tween 20. Tubes will be labeled with Unique ID and date of collection.

Tubes will be placed in a cooler for transportation to the Parsonnet Lab.

Swab samples will be logged at the Parsonnet Lab by unique ID, date of collection and initials of the person receiving them.

Swab samples will be stored at -80° for future use.

7.2.7. Kitchen countertop swabs

At each household, a 10 cm square area of the kitchen countertop will be swabbed with a cotton tip swab moistened with a solution of 0.15 M NaCl and 0.1% Tween 20. Tubes will be labeled with unique ID and date of collection.

Tubes will be placed in a cooler for transportation to the Parsonnet Lab.

Swab samples will be logged at the Parsonnet Lab by unique ID, date of collection and initials of the person receiving them.

Swab samples will be stored at -80° for future use. PCR product will be generated as above.

8. STUDY SCHEDULE

The Schedule of Assessments for the study (see Appendix C) is described in detail in the sections below.

8.1. Screening

Potential participants will be invited by the obstetric clinic staff to be contacted by STORK study staff. Study screening and enrollment will take place at the obstetric clinic. After explaining study objectives and procedures, inclusion and exclusion criteria will be applied, and eligible participants will be considered for enrollment. Potential participants may be screened/enrolled at their home if they prefer.

8.2. Enrollment/baseline

Enrollment will occur at the same time and location as screening. A test of comprehension will be administered and informed consent for study participation will be obtained. Subjects will be given contact information for the study and a copy of their informed consent. Blood [a tablespoon], urine and saliva samples will be taken and a structured interview will be administered (family structure information including household size, sibship size/birth order, etc.). Stool vials will be dispensed for later collection and return to the laboratory.

8.3. Follow-up

1. Before delivery:

   Each participant will be contacted weekly to ask about infectious conditions that she may be experiencing. In the case that amniocentesis is performed, a sample [a few drops] of amniotic fluid will be obtained for antibody determination. Early after enrollment and later in gestation (26-30 weeks and 34-38 weeks, at a scheduled clinic pre-natal visit), a small volume of blood will be obtained for antibody determination (either by finger stick [a half teaspoon] or as an additional volume in the case of a routine blood draw [a tablespoon]). A review of medical records for pre-pregnancy weight, parity history, chronic conditions, gestational diabetes and infections and other illnesses occurring during pregnancy will be performed. As part of routine care, a vaginal-rectal swab is collected at the 36 week visit; an additional swab will be collected for this study. A census of triclosan-containing products in the household will be taken.

2. At delivery:

   Cord blood will be collected [a few drops]. Chart review will be performed for the baby's Apgar scores, both birth weight and height, and complications of/at delivery.

3. After delivery:

   A. Weekly

   Each participant will be contacted weekly to assess the health status of the infant within the past week using a structured phone-based questionnaire. The mother will be asked to identify any infectious symptoms, antibiotic use, appetite level and medical care access.

   B. Monthly

Stool samples from the baby will be collected by the mother using specific kits and sent to Stanford.

C. Every four months

Study staff will visit the participant's home once every four months. At that time, a questionnaire will be administered for any updated information on changes in the household. Additionally, the baby's weight will be assessed; a heel stick will draw blood [a few drops]; saliva will be obtained; a swab will be taken of skin under the arm and a urine sample will be taken. The mother will be provided with kits for monthly stool sample collection for microbiome assessment from the baby. Similar samples will be obtained from the mother (stool, urine, saliva, skin swab). Environmental samples will be collected from under the baby's sleeping place and a counter in the kitchen, and an assessment of the cleanliness of the home will be performed. Cleaning products will be replenished at this time. The participant will receive a gift card at the end of every visit.

D. Every 6 months

Abstracts of the pediatrician's records for the child's height and weight, illnesses, treatments, vaccinations, and developmental milestones will be performed.

E. Annually

A single measure of maternal and paternal resting metabolic rate using indirect calorimetry will be taken. The child's resting metabolic rate using indirect calorimetry will be measured three times over three years, preferably at 4, 16 and 28 months of age.

At the 12-month annual visit, a blood sample will be drawn from both the mother [a tablespoon] and the baby [a teaspoon].

8.4. Final study visit

The final study visit will be the last annual visit.

8.5. Early termination visit

Reasons for early termination include:

- The study participant no longer meets inclusion/exclusion criteria;
- The study participant withdraws consent;
- The study participant is lost to follow-up;
- The study is terminated.

There is no early termination visit. Subjects may withdraw voluntarily from participation in the study at any time.

9. ASSESSMENT OF SAFETY

9.1. Definition of an adverse event (AE)

An adverse event (AE) is any untoward medical occurrence in a subject undergoing a study related procedure and believed reasonably to be caused by that study related procedure.

AEs arising from study procedures may include bruising or fainting during phlebotomy. No other study-related AEs are anticipated.

To be considered an AE, the event must occur within 72 hours of phlebotomy

9.2. Definition of a serious adverse event (SAE)

An SAE is any untoward medical occurrence that:

- Results in death.
- Is life-threatening. Any adverse experience that places the subject, in the view of the investigator, at immediate risk of death from the reaction as it occurred (i.e., it does not include a reaction that, had it occurred in a more serious form, might have caused death).
- Requires in-patient hospitalization or prolongation of existing hospitalization.
- Results in persistent or significant disability or incapacity.
- Is a congenital anomaly/birth defect.
- Requires intervention to prevent permanent impairment or damage.
- Important medical events that do not result in death, are not life-threatening, or do not require hospitalization may be considered serious adverse events when, based upon appropriate medical judgment, they might jeopardize the subject and might require medical or surgical intervention to prevent one of the outcomes listed above.

Primary health care providers caring for patients in the study are given the Stanford IRB case report form to be used during clinical examinations. Serious events are reported to the PI and the Stanford IRB on dedicated case reporting forms in accordance with Stanford University IRB policies.

There are no anticipated SAEs in this study. To be considered as an unexpected SAE, such an event must occur within 72 hours of phlebotomy.

9.3. Reporting procedures

9.3.1. Specific serious adverse event requirements

For those events meeting the previously described definition of a SAE, the completion of Stanford IRB Serious Adverse Event case report forms is required. This information will begin to be collected at the first subject encounter until one month following the last subject encounter. Specific information on where to send the forms is included in the MOP for this study (see Appendix D). All SAEs will be recorded on the appropriate Serious Adverse Event case report form and then will be reviewed by the IRB as needed.

The study PI will complete a Serious Adverse Event Form within the following timelines:

- All deaths, whether associated or not associated, and SAEs that occur one hour following blood draw will be recorded on the Serious Adverse Event Form and sent by fax within 24 hours of site awareness.
- SAEs other than death and immediately life-threatening events, regardless of relationship, will be reported via fax by the site within 72 hours of becoming aware of the event.

All SAEs will be followed until satisfactory resolution or until the PI deems the event to be chronic or the patient to be stable.

The PI will inform the NIH and the local IRBs of any deaths, SAEs, or life-threatening problems that occur in the study. The study will not utilize a Data Safety Monitoring Board (DSMB) / Safety Monitoring Committee (SMC).

9.3.2. Reporting of pregnancy

Pregnancy is a key inclusion criterion for this study. Pregnancy will not be confirmed for this study.

If an index mother becomes pregnant for a second time while enrolled in this study, the participant will continue in the study. Her new baby will not be followed. Findings from mothers once pregnant a second time will be considered separately from findings of other participants.

9.3.3. Procedures to be followed in the event of abnormal laboratory test values or abnormal clinical findings

Collection of laboratory data will be limited to those laboratory parameters that are relevant to study outcome measures and/or clinical outcome. Laboratory data collected within this study include levels of: TNF, grehlin, insulin, leptin, IFN-gamma, IGF, glucagon, resistin, plasminogen activator inhibitor 1 (*PAI-1*), IL10, CRP, ESR, LPS and triclosan; additionally, both microbial antibodies and microbial diversity will be assessed.

Because treatment is not provided as part of this study, no laboratory measures of treatment safety are conducted and no toxicity tables will be defined.

9.3.4. Type and duration of the follow-up of participants after adverse events

There are no SAEs anticipated from phlebotomy. Minor adverse events such as fainting or bruising at the sight of phlebotomy may occur. As with all AEs, subjects are given our research office number to call should they be concerned about such as event. All subjects will be called within 24 hours following any report of adverse events and medical follow-up identified as necessary. Follow-up will continue until the AE is resolved.

9.4. Halting rules for the protocol

Given the essentially observational nature of this study, we *a priori* expect no SAEs. When identified, SAEs are reviewed in real time by the PI. One serious related SAE will trigger a suspension of the study by the PI and review of all AEs that have occurred in the study, with particular focus on the SAE. Such reviews may result in no protocol change, in precautionary decisions (including modification of consent forms), or in suspension of the protocol if necessary. Greater than one related SAE will be strongly considered as grounds for halting the study.

Non-serious AEs will be reviewed by the PI on a weekly basis and a determination as to the relation of the event to study procedures will be made. In addition to review of adverse event reports on receipt, the IRB will review all adverse event case reports at their annual protocol review.

Other reasons for halting the study may include:

- New information that renders the protocol unethical;
- Failure to meet enrollment targets after remedial steps are taken;
- Changes in research priorities or funding constraints of the PI or the NIH.

It is possible that the intervention aspect of the study could be halted, leaving the cohort aspect ongoing. Reasons for halting the intervention aspect include:

- New information that renders the study of triclosan unethical;
- An inability to detect any difference in triclosan levels between households;
- Changes in research priorities or funding constraints of the PI or the NIH.

9.5. Premature withdrawal of a participant

Participants have the right to stop participation in the study at any time. In the case of premature withdrawal of a participant from the study, all attempts will be made to complete the termination visit for the mother / infant pair.

Participants who cannot be located after two months will be dropped from the study.

9.6. Replacement of a participant who discontinues study treatment

- If a participating mother drops out while the study is still enrolling participants, or prior to the birth of her child then she will be replaced.
- If a participating mother/child pair drops out within two months of the final cohort participant being enrolled, then she will be replaced.
- If a participating mother/child pair drops out after three months of the final cohort participant being enrolled, then the pair will not be replaced.

10. CLINICAL MONITORING STRUCTURE

10.1. Site monitoring plan

The PI is responsible for day-to-day monitoring of the study activities. She will review all study activities, recruitment objectives, protocol compliance, and data and document integrity with study personnel on a weekly basis and will be available for questions and problems at all times. She will ensure that informed consent is appropriately obtained from all subjects. Additionally, she will see that resources are appropriately allocated and that study personnel are adequately supervised and supplied.

The PI will be responsible for ensuring all appropriate communications—including data records and reports—are transmitted to the IRBs and co-investigators in a timely manner so that such monitoring can be conducted. The PI will also be responsible for ensuring that monitoring findings of these bodies that require action are addressed.

The study will be carried out at Stanford University supported by collaborative referral arrangements with clinics participating in the study. Recruitment procedures will be approved by the Stanford University IRB and the SCVMC IRB. Human subjects protections are ensured through annual IRB reviews at Stanford and at SCVMC. Although recruitment sites will be used, only Stanford personnel will recruit subjects, collect study information and manage samples and data. Health care providers at the clinic sites will refer appropriate subjects to the Stanford personnel for recruitment. To keep personnel at the recruitment sites informed, an in-service workshop will be conducted at each recruitment site. Provider orientation materials will be developed. Standardized recruitment forms will be developed. Semi-annual meetings will be scheduled with each site to report study progress and to discuss any study related issues with recruitment and follow-up arrangements.

Study staff will be present at participating clinics at least one day per week to trouble-shoot as well as to follow-up on recruitment.

10.2. Safety monitoring plan

The PI will review all AEs and will be responsible for identifying appropriate medical resources to respond to AEs as needed.

No Data Safety and Monitoring Board (DSMB) or Safety Monitoring Committee (SMC) is anticipated for this study.

11. STATISTICAL CONSIDERATIONS

11.1. Overview and study objectives

This cohort study of 200 infants followed from in *utero* to age 3 years has the primary objective of determining the association between infectious disease load, as measured by cumulative sick-days, and growth, as measured by height-for-weight Z-score.

A randomized intervention is nested within the cohort. It has the primary objective of determining whether exposure to triclosan, as measured by intervention arm, decreases infectious disease load.

Accounting for a 20% drop-out rate per year, we expect that 103 children will complete the full three years of the cohort study.

Secondary analyses for the cohort include:

- Assessment of the association between infection and microbial diversity, by characterizing the microbiota by 16S rRNA gene sequence analysis.
- Assessment of the association between infection and the breadth of the developing immune system response, as measured by the types and proportions of naïve, effector memory and central memory T cells.
- Assessment of the association between growth and microbial diversity.
- Assessment of the association between growth and breadth of the developing immune system response.

Secondary analyses for the intervention include:

- Assessment of the association between triclosan exposure and microbial diversity.
- Assessment of the association between triclosan exposure and the breadth of the developing immune system response..

Exploratory analyses encompass a wide range of topics, with several examples listed below.

- Association between specific chronic infections (e.g., the various herpesviruses and *H. pylori*) and weight gain.
- Evaluation of the sequence of infection acquisition and subsequent cytokine responses to other infectious exposures.
- Correlation between the frequency of antibiotic use and growth.
- Association between infection and metabolic rate, acutely and chronically.

- Evaluation of the duration of maternal antibodies in infants. [Although common wisdom maintains that antibodies persist for six months, the actual data on maternal antibodies are few, varied and only describe a small number of infectious diseases.]
- Description of the epidemiology of childhood infections. [Surprisingly, the incidence of many infections in childhood, including skin infections, streptococcal pharyngitis, diarrheal diseases, and many other common illnesses is largely unknown.]
- Evaluation of the associations between infection and other common childhood diseases such as asthma and juvenile onset diabetes.
- Evaluation of the effect of an episode of fever on the microbiome.

A wide variety of clinical measures will have been collected in this study, with each being either a primary exposure measure, a primary outcome of interest, a potential confounder of the relationships being considered, or a component of the metabolic pathways being explored.

- Exposure measures include: reports of infectious disease-like symptoms, temperature; microbial seroconversions (number and organism); age at onset and chronicity of specific infections, sequence of infection acquisition
- Outcome measures include: interval change in anthropomorphic measures between visits
- Confounders include: diet, activity, obesity in the family, family structure;
- Elements of the etiologic pathway include: metabolic rate, adipocytokines, cytokines.

The analytic role of these measures is specified in Table 2 below.

Table 2: Clinical measures

**Time and frequency**	**Who tested**	**Data to be obtained**	**Role in analysis**
PRENATAL			
Once	Family	Anthropometric measurements	Confounder
Once	Family	Family structure (household size, sibship size/birth order, etc.)	Confounder
Once	Family	Socioeconomic status	Confounder
Once	Mother	Serum sample for microbial antibodies	Primary exposure
Once	Mother	Blood sample for TNF, grehlin, insulin, leptin, IFN-gamma, IGF, glucagon, resistin, PAI-1, IL10, IL17, CRP, ESR	Possible pathogenic pathway
Once	Mother	Stool/gingival samples for microbial DNA extraction	Outcome

Table 2: Clinical measures

Time and frequency	Who tested	Data to be obtained	Role in analysis
Once	Mother	If obtained, amniotic fluid for microbial DNA extraction	Primary exposure
Weekly	Mother	Infectious diseases symptoms and treatments	Primary exposure
Twice in prenatal period	Mother	Finger stick blood sample for microbial antibodies	Primary exposure
Each prenatal visit	Mother	Medical record abstract for weight	Confounder
Each prenatal visit	Mother	Medical record abstract for infections, gestational diabetes and other illnesses	Primary exposure
DELIVERY			
		Apgar scores	Confounder
		Birth weight and height	Confounder
		Cord blood for antibody determination	Primary exposure
POST NATAL			
Weekly	Child	Infectious diseases symptoms and treatments	Primary exposure
Weekly	Child	Appetite (normal, low)	Metabolic pathway
Each well baby visit	Child	Anthropometric measurements	Outcome
Each well baby visit	Child	Developmental milestones	Confounder
Every 4 months	Child	Weight	Outcome
Every 4 months	Child	Urine	Primary exposure
Every 4 months	Child	Heel stick blood for microbial antibodies	Primary exposure
Every 4 months	Family	Change in family structure	Confounder
Every 4 months	Family	Participation in out of home care	Confounder
Every 6 months	Child	Abstracts of physician records for medical illnesses and treatments	Primary exposure
Monthly	Child	Stool samples for microbial flora	Outcome
Annually	Child	Child's blood for TNF, grehlin, insulin, leptin, IFN-gamma, IGF, glucagon, resistin, PAI-1, IL10, IL17, CRP, ESR, LPS	Metabolic pathway

Table 2: Clinical measures

Time and frequency	Who tested	Data to be obtained	Role in analysis
Annually	Child	Sleeping or resting metabolic rate through indirect calorimetry	Metabolic pathway
Annually	Mother	Urine	Confounder
Single reading		Maternal and paternal resting metabolic rate	Confounder

11.2. Analysis plan

A study analysis plan will be written prior to database finalization and close.

All testing will be 2-tailed, and assume both a confidence of 95% and a power of 80%. All distributions will be tested for normality; non-parametric testing will be used in the case of non-normality.

11.2.1. Cohort

Our primary hypothesis is that the distribution of height-for-weight Z-scores is not different between the high and low infectious disease load groups.

- *Outcome*: Z-score
- *Predictor*: Days of illness, categorized into high and low load based on the distribution of sick-days.
- *Analysis*: T-test for 2 groups, using multiple regression to look at potential confounders and effect modifiers.
- *Diagnostic to validate the Z-score approach*: A random intercept model.

Descriptive statistics will be provided for infectious disease load, for specific combinations of infections, and for individual infections. Incidence and seroconversion for overall and specific infections will be presented over a variety of time points. Statistics on age at onset and chronicity of specific infections will be calculated, and the sequence of acquisition of infections will be generated.

Descriptive statistics will be provided for the primary and other growth variables, including height-for-weight, height-for-age and weight-for-age. Comparison of summary Z-score statistics of the sample to the appropriate WHO reference population will be performed.

Descriptive statistics for each of the immune response variables and appropriate combinations will be provided.

Sequences generated from pyrosequencing barcoded 16S rRNA gene PCR amplicons will be analyzed using the open source software package Quantitative Insights Into Microbial Ecology (QIIME)(37). 16S rRNA gene sequences will be assigned to operational taxonomic units (OTUs) using UCLUST (38) with a threshold of 97% pair-wise identity, then classified taxonomically using the Ribosomal Database Project (RDP) classifier 2.0.1 (39). For tree-based analyses, a single representative sequence for each OTU will be

aligned using PyNAST (40), then a phylogenetic tree will be built using FastTree (41). The phylogenetic tree will be used for measuring the a-diversity (phylogenetic diversity, PD (42)) and b-diversity (using unweighted UniFrac (43))) of samples.

The "nearest shrunken centroid" method will be used to identify OTUs that are specifically over (or under)-represented in a given category (44). The amount of shrinkage will be chosen in order to minimize the overall misclassification error. The analysis will be performed using the PAM-R (Predictive Analysis of Microarrays) package under R software.

Associations between infectious disease load, growth, immune response and microbiome response will be considered, taking into consideration confounders and etiologic pathways as appropriate.

Subjects who are lost to follow-up will be included in time series analyses until censored. Factors associated with loss-to follow-up will be evaluated for systematic biases. Subjects who drop out will be replaced in the rolling recruitment until 2 months after the last participant is enrolled.

We will examine patterns of missing data to identify potential systematic biases. Sensitivity analyses will be conducted to assess the influence of missing data on study results.

11.2.2. Intervention

Analysis will be by intended allocation (intent-to-treat). No per-protocol analyses are planned.

Cumulative incidence and incidence density for infectious disease load over time will be calculated; additional analyses will consider specific types or groupings of disease (e.g., upper respiratory vs. diarrheal; specific infections). Cox regression will be used to compare the intervention arms while taking time into consideration and adjusting for potential confounders or effect modifiers (e.g., crowding, day-care exposure, etc.).

The association between study arm and microbial diversity will be explored, as will the association between study arm and immune system development, as in Section 11.2.1 above.

11.3. Sample size considerations

11.3.1. Cohort

Precise data regarding infectious disease burden or load are rare. Upper respiratory infections (URI) in infants and toddlers are thought to occur approximately 6-10 times per year, each lasting approximately 1 week, for an estimated 56 (42-70) days with URI per year. Diarrheal disease has been shown to "last less than 2 weeks" for 4.5% of child-care days, or approximately 15 affected days per year. This leads to a cumulative total (URI and diarrhea) of 71 (57-85) days of illness per year.

We will assume that high infectious disease load is the median or more days of illness per year, and low infectious disease load is one day less or fewer than the median days of illness per year.

We will assume that the reference population generated by the WHO, from which the Z-scores are generated, is based on a population where infectious disease load is relatively high. Thus our sample with high infectious disease load will have a mean Z-score of 0. We assume that the sample with low infectious disease will have an increased mean Z-score compared to this reference population. Given an alpha of .05 and power of .8, then (Table 3):

- A sample of 100 pairs would be sufficient to detect a 40% difference in Z-score, assuming a common standard deviation of 1.
- 64 subjects per arm would be sufficient to show a 10% difference in Z-score with a standard deviation of .2.

Table 3: Total sample size of pairs required for varying effect sizes

Z-score in high load group	Z-score in low ID load group	SD	Effect size	N per group
0	0.40	1	0.4	100
0	0.2	0.4	0.5	64
0	0.1	0.2	0.5	64
0	0.1	0.25	0.25	100

*Alpha = 0.05, power = .8
** Standard deviation

11.3.2. Intervention

We will assume that over one year, triclosan exposure will cause a 20% difference in number of sick-days as described above. Given an alpha of .05 and power of .8, then (Table 4):

- If infants in non-triclosan households are sick for 71 (SD = 20) days over one year, then a total of 33 subjects in each arm will be sufficient to detect a 20% decrease from triclosan exposure.
- 100 subjects in each arm will allow us to see a 20% decrease in sickness days attributable to triclosan over one year if infants in non-triclosan households are sick for 60 (SD = 30) or 50 (SD = 25) days.

Table 4: Total sample size* required based on a 20% difference in sick-days, considering different numbers of total sick-days and its deviation.

**Sick days in non-triclosan arm**	**Sick days in triclosan arm**	**Standard deviation (SD)****	**Total sample size**
71	57	20	66
71	57	30	146
50	40	20	126

Table 4: Total sample size* required based on a 20% difference in sick-days, considering different numbers of total sick-days and its deviation.

Sick days in non-triclosan arm	Sick days in triclosan arm	Standard deviation (SD)**	Total sample size
50	40	25	198
50	40	30	284
60	48	30	198

*Alpha = 0.05, power = .8
**SD: equal in both arms

11.4. Maintenance of trial treatment randomization codes

Randomization of pregnant women to the two arms of cleaning products (either containing no triclosan or containing triclosan) will occur at enrollment. The School of Medicine's Data Coordinating Center (DCC), which specializes in the planning, development, management, and operation of systems for studies and trials, has generated a web-based program that performs biased coin randomization, allowing the equal distribution across trial arms of, in this case, (1) first born children; (2) non-first-born children. Study staff will access this program at the time of enrollment; full documentation is maintained by the DCC.

12. QUALITY CONTROL AND QUALITY ASSURANCE

The study will be conducted in accordance with procedures identified in the protocol.

Paper forms and laboratory reports will be reviewed by a study supervisor, approved, and initialed prior to data entry. Electronic forms will be reviewed, approved and initialed with an electronic signature.

The PI will maintain a Manual of Procedures (MOP) covering site recruitment, screening, eligibility criteria, data sources, laboratory methods, analyses and study-specific protocols (see Appendix D, MOP). Modifications will be documented by date, rationale, and nature of modification. The MOP will be used to orient and train all study staff, who will function under the supervision of a study coordinator. All field and laboratory personnel will use standard operating procedures (SOPs) for managing samples and data.

Data will be evaluated for compliance with the protocol and accuracy in relation to source documents.

Weekly meetings of the PI and study staff will be scheduled and used for troubleshooting and refinement of specific procedures. Orientation meetings will be held at each referral site, with in-service meetings scheduled at least semi-annually. Forms and procedures will be reviewed.

Data will be evaluated for compliance with protocol and accuracy in relation to source documents. All documents will be piloted, and during the first three months of the study, fields will be checked for consistent interpretation and coding. Semi-annually thereafter, a 10% random sample of fields will be selected for trace-back from data based information to hard copy, and all fields will be checked under the supervision of the study

coordinator. SQL queries will be run periodically, with feedback on anomalies or missing data.

Appropriate controls will be utilized for all laboratory assays. Every six months, blinded control samples will be submitted for QA/QC testing. Laboratory notebooks will be reviewed every six months.

Freezers for storage of samples will be monitored daily.

13. ETHICS / PROTECTION OF HUMAN SUBJECTS

13.1. Declaration of Helsinki

The PI will ensure that this study is conducted in full conformity with the current revision of the Declaration of Helsinki, or with the International Conference for Harmonisation Good Clinical Practice (ICH-GCP) regulations and guidelines, whichever affords the greater protection to the subject. Consent procedures conform to the Declaration of Helsinki in accordance with standards of Stanford University for research involving human subjects.

13.2. Participant confidentiality

Subject confidentiality is held strictly in trust by the PI, her staff, the recruiting clinics and the sponsor(s) and their agents. This confidentiality is extended to cover testing of biological samples and genetic tests in addition to the clinical information relating to participating subjects.

The study data and all other information generated will be held in strict confidence. No information concerning the study data will be released to any unauthorized third party without prior written approval of the Stanford University IRB and the NIH.

Authorized representatives of the NIH may inspect all documents and records required to be maintained by the PI, including but not limited to, Primary Provider Medical Records (office, clinic or hospital) for the subjects in this study. Subjects are informed prior to consent of institutions, authorities, and individuals to whom data may be made available. No information concerning the data will be released to any other (unauthorized) third party without prior written approval of the PI and the subject.

Subjects are informed prior to consent of the nature of personal information that may be obtained from medical charts and laboratory testing. They are also informed that their samples may be stored following completion of the study for future, related research, but that their name will be forever unlinked to their samples. In addition, subjects are informed that results of research studies are not available to them or other parties, and do not become part of their Primary Practitioner Medical Record. HIPAA authorizations will be approved by the IRB at Stanford University (included in the Appendix as part of the consent form).

All subjects are assigned a unique, anonymous study ID upon referral that shall serve as a key index for location of all subject records and specimen samples. Databases are password-protected.

13.3. Institutional review board/medical ethics committee

Stanford University's Institutional Review Board, (called the "Administrative Panels on Human Subjects in Medical Research"; see http://humansubjects.stanford.edu) has reviewed, and will continue to review the protocol, the protocol amendments, the adverse event reports, and the annual updates for this proposal. All amendments to the protocol or consent materials will be approved before they are placed into use. The IRB is registered with the OHRP.

13.3.1. Assent or informed consent process (in case of a minor)

Women are eligible for enrollment only if they are aged 18 years or greater, so no mother will be considered a minor. These same women will provide consent for their infants to be in the study. At the current time, the study will follow infants only until the age of three years.

13.4. Exclusion of women, minorities and children (special populations)

Special populations participating in this study include pregnant women, women of Hispanic origin, and infants and so exclusion is not required. We will limit enrollment to individuals with whom we can communicate effectively in English or Spanish (see MOP).

13.5. Informed consent process

Informed consent is a process that is initiated prior to the individual's agreeing to participate in the study and continuing throughout the individual's study participation. Discussion of risks and possible benefits of participation in this study will be provided to the subjects. A consent form describing in detail the study procedures and risks will be given to the subject and written documentation of informed consent is required prior to enrollment in the study. Consent forms will be approved by the IRB and the subject will be asked to read and review the document. Upon reviewing the document, the investigator will explain the research study to the subject and answer any questions that may arise. Following review of the informed consent, subjects are asked to take a brief test of comprehension (see Appendix A4); any misunderstandings are then reviewed and discussed. The subjects will have the opportunity to take an unsigned form home to discuss with family or others or to think about it prior to agreeing to participate. The subjects will sign the informed consent document prior to being enrolled in the study.

The subjects may withdraw consent at any time throughout the course of the study.

A copy of the informed consent document will be given to the subjects for their records.

The rights and welfare of the subjects will be protected by emphasizing to them that the quality of their medical care will not be adversely affected if they decline to participate in this study.

Consent will be administered in a language in which the subject is proficient (either English or Spanish).

Consent occurs at the time of enrollment. Subjects are informed that, if they meet enrollment criteria, they will be enrolled in the cohort study and randomized to an intervention arm. Thus, by signing this consent, they are enrolling to provide samples, have their samples tested, to have some samples conserved for possible future study, to be followed up twice during pregnancy, to be followed after the birth of their child for up to three years, to be contacted weekly after the birth of their child to provide health status information, to provide samples (blood, stool, saliva, urine, skin swabs) over 11 total visits from the child and to be tested by indirect calorimetry (mother, father if possible, and the child). In addition, they are asked to give permission for investigators to obtain selected information from both their and their child's Primary Provider Medical Record pertaining to assessments done by their clinician and their pediatrician, for infectious disease and other diagnoses and treatment, height and weight gain, developmental milestones and vaccination status. This consent additionally does ask permission to re-contact the subject about further add-on studies.

In the consent form, participants are given the opportunity to choose whether their specimens may be kept for future unspecified research, unlinked to their name or other identifying information.

The current informed consent form is in Appendix A3.

13.6. Study discontinuation

Participants are free to discontinue participation in the study at any time. All efforts will be made to understand why the participant has chosen to discontinue participation.

The study may be discontinued at any time by the PI, the NIH, or the Stanford University IRB.

13.7. Future use of stored specimens

Residual specimens will be maintained after the study is complete if the subject so consents (see Appendix A3, Consent form). Stored specimens will be unlinked from personal identifiers after completion of the study. Samples will be stored at the Stanford University School of Medicine. Specimens not selected for use may be stored up to Year 2035 for use by investigators in related investigations. All future studies will be reviewed by the Stanford University IRB.

14. DATA HANDLING AND RECORD KEEPING

Data will be managed as below. Copies of all datasets will be provided to the NIH at completion of the study and at any time requested by the NIH.

14.1. Data management responsibilities

The PI will maintain complete and accurate documentation for the study.

The study will employ a Study Coordinator, a Data Manager/Analyst and an Epidemiologist/Statistician, all of whom will, under supervision of the PI, be responsible for development of record-keeping systems, quality audits, and preparation of analytic datasets.

For each subject, a hard copy file of source material, which will be maintained at the study office (S131, Grant Building, Stanford School of Medicine), will be filed by unique ID. Any source material from Primary Provider Medical Records, if not provided in electronic format, will be photocopied by study personnel. Source material includes:

- Recruitment form
- Interview responses
- Clinical laboratory reports
- Medical chart abstracts
- A sample tracking form
- A provider notification sheet

A log sheet at the front of each file will document receipt of data from different sources and any actions taken. A contact sheet containing essential contact information and a copy of consent forms is kept in a locked cabinet in the office of the Study Coordinator. Source material is reviewed upon receipt by the Study Coordinator and initialed for consistency and correctness prior to database entry. Data are entered into an electronic database (MS ACCESS or REDCap) using coded forms with range and logical checks per field. Forms, coding dictionary, and instructions for use of the electronic databases will be contained in the MOP.

Laboratory research studies will include results from routine blood tests, cytokine panels, assessment of antibodies, and triclosan levels in urine. Laboratories conducting research studies will provide output on Excel spreadsheet templates, which are emailed to the PI and the Study Coordinator after each run for review. These forms also document control standards, standard curves, and reliability statistics. Following review, the data are compiled by the study Data Manager/Analyst for import into the study database. Plate lay-out sheets are maintained by each laboratory in dedicated notebooks and can be used for reconciliation as questions arise. A line list is generated and pasted into an Excel spreadsheet for transmittal, review, and entry into the database. Laboratory performance statistics, including standard curve parameters and R-squares, are logged in files linked to each batch run. Periodically, these are plotted over time for detection of drift.

The laboratories performing the analysis of the microbiome will provide output as raw data files. A record of which files have been received will be kept.

The PI or project Data Manager/Analyst will be responsible for transmitting copies of the electronic database to funding and oversight authorities as required at the end of study.

14.2. Data capture methods

Weekly health status updates are collected over the telephone as an automated telephone poll or in person. Results from the latter are entered directly into REDCap and from the former are automatically collected as a CVS file.

Interviewer administered questionnaire responses are entered onto an electronic form using a tablet, with these electronic data then transferred into REDCap; if entered on paper forms, double data entry will be used to enter data into REDCap.

Information obtained from medical chart reviews, if not received as an electronic file, is documented on standardized paper forms, and then deposited in the subject's file after review by the Study Coordinator.

Clinical laboratory reports, including reference ranges and individual results, are documented electronically by the Stanford Clinical Laboratories or another reference laboratory with hard-copy forms sent to the PI.

All clinical laboratory results are faxed to the study office on individualized standard report forms as completed.

Serologic and cytokine assays are run in batch with results captured automatically on Excel spreadsheet templates and emailed to the PI, Data Analyst and Study Coordinator for review.

Results of microbiome analysis are captured automatically off the sequencers as raw .sff files, which are then run through a bioinformatics pipeline (Google QIIME).

14.3. Types of data

The following data types will be collected:

- Interview responses (close-ended with comment fields)
- Clinical data (close ended with comment fields)
- Medical chart abstract fields (close-ended with comment fields)
- Clinical laboratory reports from the Stanford Clinical Laboratory and other reference laboratory (standard report form by test, quantitative and qualitative result, and laboratory reference range)
- Laboratory (pulmonary function) output for indirect calorimetry (Excel spreadsheet template containing test standards, measurement of CO2 production, respiratory exchange ratio)
- Laboratory (immunology) output for antibody detection and quantification (Excel spreadsheet template, either by standard, antigen, and quantitative result or by array format)
- Laboratory (CDC) output for triclosan levels
- Sequencer output for microbiome findings

14.4. Source documents and access to source data/documents

All subjects will be assigned a unique anonymous study ID at the point of enrollment. Each participant's baby will be given a related ID. The code for these numbers will be retained under the strictest confidentiality. All questionnaires, laboratory specimens, and electronic records will be indexed with this ID number. A single research employee (the Study Coordinator) and the PI will have access to the code and will not disclose the code to any other research personnel, except by task-specific authorization, such as to enable field staff to identify medical records for chart reviews, or to place follow-up phone calls to participants. The codes that link the name of the participant and the study ID will be kept in a locked cabinet within the research space of the PI. All data gathered from study subjects to be included in the analysis will reference only the ID number and will not contain personal identifiers. Each subject's study information will remain confidential among the investigators and research personnel at all times.

Authorized representatives of regulatory agencies will be permitted to examine (and, when required by applicable law, to copy) records for the purposes of quality assurance reviews, audits and evaluation of the study safety and progress.

Documents for the study include a hard copy subject file, blood, saliva, urine and stool specimens, documentation and reports of laboratory tests. The hard copy file will contain:

A. Information containing personal identifiers

- Recruitment form
- Consent form
- Contact information
- Adverse event case report form (as needed)
- Serious adverse event report form (as needed)
- Primary provider and pediatrician medical chart abstraction (periodic).

B. De-identified information

- Questionnaires
- Laboratory reports (routine, antibodies and microbiome)
- Participant notices (de-identified)
- Provider notices (de-identified)

Hard copy files are kept in a locked file within the research office in the Grant Building, Room S131, at the Stanford Medical School under supervision of the Study Coordinator. Access is restricted to authorized study personnel and requires permission of the Study Coordinator. Information which contains personal identifiers is kept in a locked file that is separate from all other study source documents.

In addition to hard copy files, specimen bank accounts and batch laboratory output will be prepared on Excel spreadsheet templates, indexed by subject code and dates. These are filed in a dedicated laboratory notebook by the study's laboratory director, and are

available for uploading to an electronic database following review by the Study Coordinator.

The study will create a web-based relational database (HTML-DB) and also use the REDCap biomedical study database development platform for participant contact information, data entry, audit, and retrieval of research data at the subject, time, and laboratory specimen level. The databases will be maintained with restricted access privileges for different research personnel. Data entry will be governed by a code book and MOP. To reduce coding errors and data management costs, interviewer-administered questionnaire responses will be entered directly into an electronic database and approved for accuracy by the participant. To minimize errors, other data entry will be performed twice and then compared. The Data Manager/Analyst will develop range and consistency check programs to clean the data. Data will be analyzed in SAS (version TBD).

14.5. Timing/reports

Individual laboratory reports are not needed for the study to proceed (laboratory tests, antibody assessment, microbiome); all testing can be performed in a batched fashion either at the end of the study or as convenient.

No affiliated laboratories receive information regarding subject randomization status; their results are thus blinded to study arm.

Final analysis points are currently set at Year 5 (final follow-up).

Semi-annual reports will be prepared documenting study progress, including number referred, eligible, enrolled, withdrawn, and completing.

14.6. Study records retention

Study files, except for future use consent forms, will be maintained for a minimum of three years after completion of study data collection and analysis; the database and analytic datasets may be stored indefinitely as electronic data.

14.7. Protocol deviations

Analyses for the intervention will follow only an intent-to-treat format. Participants in the non-triclosan arm who report the consistent use of triclosan-containing household or personal cleaning products, or those in this arm who demonstrate high urinary or blood concentrations of triclosan, will be included in the intent to treat analysis as initially randomized. Protocol deviations will not be tabulated.

14.8. Future use of stored specimens

Residual specimens will be maintained after the study is complete if the subject so consents (see Appendix A3, Consent form). Stored specimens will be unlinked from personal identifiers after completion of the study. Samples will be stored at Stanford University School of Medicine. Specimens may be stored up to Year 2035 for use by

investigators in related investigations. All future studies will be reviewed by the Stanford University IRB.

15. PUBLICATION POLICY

Key findings from this study, as well as study design, are expected to be made public at the end of the study. Authors of publications will include the PI and collaborators. In addition, investigators representing referring clinics will be offered the opportunity to participate in manuscript development and authorship. Individual study subjects will not be identified, nor will they be identifiable in any publication or presentation.

16. SCIENTIFIC REFERENCES

1. Centers for Disease Control and Prevention. Overweight and Obesity. 2008. (Accessed December 8, 2008, at http://www.cdc.gov/nccdphp/dnpa/obesity/index.htm.)

2. Wilkin TJ, Voss LD, Metcalf BS, Mallam K, Jeffery AN, Alba S, Murphy MJ. Metabolic risk in early childhood: the EarlyBird Study. Int J Obes Relat Metab Disord. 2004 Nov;28 Suppl 3:S64-9.

3. Kamath CC, Vickers KS, Ehrlich A, McGovern L, Johnson J, Singhal V, Paulo R, Hettinger A, Erwin PJ, Montori VM. Clinical review: behavioral interventions to prevent childhood obesity: a systematic review and metaanalyses of randomized trials. J Clin Endocrinol Metab. 2008 Dec;93(12):4606-15. Epub 2008 Sep 9.

4. Ionescu AA, Nixon LS, Shale DJ. Cellular proteolysis and systemic inflammation during exacerbation in cystic fibrosis. J Cyst Fibros. 2004 Dec;3(4):253-8.

5. Garza C. Effect of infection on energy requirements of infants and children. Public Health Nutr. 2005 Oct;8(7A):1187-90.

6. Benhariz M, Goulet O, Salas J, Colomb V, Ricour C. Energy cost of fever in children on total parenteral nutrition. Clin Nutr. 1997 Oct;16(5):251-5.
7. Torine IJ, Denne SC, Wright-Coltart S, Leitch C. Effect of late-onset sepsis on energy expenditure in extremely premature infants. Pediatr Res. 2007 May;61(5 Pt 1):600-3.

8. Lane BJ, Provost-Craig MA. Resting energy expenditure in asymptomatic HIV-infected females. J Womens Health Gend Based Med. 2000 Apr;9(3):321-7.

9. Awad elKarim MA.The effect of schistosomiasis on 24-hour energy expenditure. J Trop Med Hyg. 1986 Dec;89(6):303-7.

10. Aquilani R, Boschi F, Contardi A, Pistarini C, Achilli MP, Fizzotti G, Moroni S, Catapano M, Verri M, Pastoris O. Energy expenditure and nutritional adequacy of

rehabilitation paraplegics with asymptomatic bacteriuria and pressure sores. Spinal Cord. 2001 Aug;39(8):437-41.

11. Utaka S, Avesani CM, Draibe SA, Kamimura MA, Andreoni S, Cuppari L.Inflammation is associated with increased energy expenditure in patients with chronic kidney disease. Am J Clin Nutr. 2005 Oct;82(4):801-5.

12. Piche T, Schneider SM, Tran A, Benzaken S, Rampal P, Hébuterne X. Resting energy expenditure in chronic hepatitis C. J Hepatol. 2000 Oct;33(4):623-7.

13. Pacifico L, Anania C, Osborn JF, Ferrara E, Schiavo E, Bonamico M, Chiesa C. Long-term effects of Helicobacter pylori eradication on circulating ghrelin and leptin concentrations and body composition in prepubertal children. Eur J Endocrinol. 2008 Mar;158(3):323-32.

14. Hube F, Hauner H. The role of TNF-alpha in human adipose tissue: prevention of weight gain at the expense of insulin resistance? Horm Metab Res. 1999 Dec;31(12):626-31. Review.

15. Guyer B, Freedman MA, Strobino DM, Sondik EJ. Annual summary of vital statistics: trends in the health of Americans during the 20th century. Pediatrics. 2000 Dec;106(6):1307-17.

16. Charles J, Pan Y, Britt H. Trends in childhood illness and treatment in Australian general practice, 1971-2001. Med J Aust. 2004 Mar 1;180(5):216-9.

17. Vargas MH. Ecological association between scarlet fever and asthma. Respir Med. 2006 Feb;100(2):363-6. Epub 2005 Jun 6.

18. Ilyas M, Tolaymat A. Changing epidemiology of acute post-streptococcal glomerulonephritis in Northeast Florida: a comparative study. Pediatr Nephrol. 2008 Jul;23(7):1101-6. Epub 2008 Mar 29.

19. Phares CR, Lynfield R, Farley MM, Mohle-Boetani J, Harrison LH, Petit S, Craig AS, Schaffner W, Zansky SM, Gershman K, Stefonek KR, Albanese BA, Zell ER, Schuchat A, Schrag SJ; Active Bacterial Core surveillance/Emerging Infections Program Network. Epidemiology of invasive group B streptococcal disease in the United States, 1999-2005. JAMA. 2008 May 7;299(17):2056-65.

20. Walker-Smith J. Paediatric gastroenterology 1966-2000. Clin Med. 2008 Jun;8(3):292-5. Review.

21. Kang JY, Hoare J, Majeed A, Williamson RC, Maxwell JD. Decline in admission rates for acute appendicitis in England. Br J Surg. 2003 Dec;90(12):1586-92.

22. Zhou F, Shefer A, Kong Y, Nuorti JP. Trends in acute otitis media-related health care utilization by privately insured young children in the United States, 1997-2004. Pediatrics. 2008 Feb;121(2):253-60.

23. Sanz Y, Santacruz A, Gauffin P. Gut microbiota in obesity and metabolic disorders. Proc Nutr Soc. 2010 Aug;69(3):434-41. Epub 2010 Jun 14.

24. Ley RE, Turnbaugh PJ, Klein S, Gordon JI. Microbial ecology: human gut microbes associated with obesity. Nature. 2006 Dec 21;444(7122):1022-3

25. http://en.wikipedia.org/wiki.Triclosan. Accessed 3/3/11

26. http://www.fda.gov/forconsumers/consumerupdates/ucm205999.htm. Accessed 3/3/11

27. Bhargava NH, Leonard PA, Triclosan: Applications and safety. Am J Infect Control 1996; 24:209-18

28. Rodricks JV, Swenberg JA, Borzelleca JF, Maronpot RR, Shipp AM. Triclosan: A critical review of the experimental data and development of margins of safety for consumer products. Critical Reviews in Toxicology May 2010, Vol. 40, No. 5, Pages 422-484: 422-484.

29. Jacobs MN, Nolan GT, Hood SR. Lignans, bacteriocides and organochlorine compounds activate the human pregnane X receptor (PXR).Toxicol Appl Pharmacol. 2005 Dec 1;209(2)

30. Paul KB, Hedge JM, DeVito MJ, Crofton KM. Short-term exposure to triclosan decreases thyroxine in vivo via upregulation of hepatic catabolism in Young Long-Evans rats.Toxicol Sci. 2010 Feb;113(2):367-79. Epub 2009 Nov 12.

31. Palenske NM, Nallani GC, Dzialowski EM. Physiological effects and bioconcentration of triclosan on amphibian larvae. Comp Biochem Physiol C Toxicol Pharmacol. 2010 Aug;152(2):232-40. Epub 2010 Apr 22

32. Witorsh RJ, Thomas JA. Personal care products and endocrine disruption: a critical review of the literature. Crit Rev Toxicol, 2010; 40(S3): 1-30

33. www.clinicaltrials.gov; data viewed on 1/4/11

34. McConeghy KW, Mikolich DJ, LaPlante KL. Agents for the Decolonization of Methicillin-Resistant Staphylococcus aureus. Pharmacother 2009;29(3):263–280

35. Aiello AE, Larson EL, Levy SB. Consumer Antibacterial Soaps: Effective or Just Risky? Clin Infect Dis. (2007) 45 (Supplement 2): S137-S147.

36. http://www.who.int/nutgrowthdb/about/introduction/en/index4.html. Accessed 3/3/11.

37. Caporaso, J. G., J. Kuczynski, et al. (2010). "QIIME allows analysis of high-throughput community sequencing data." Nat Methods **7**(5): 335-6.

38. Edgar RC. Search and clustering orders of magnitude faster than BLAST. Bioinformatics 2010 Oct 1;26(19):2460-1.

39. Wang, Q., G. M. Garrity, et al. (2007). "Naive Bayesian classifier for rapid assignment of rRNA sequences into the new bacterial taxonomy." Appl Environ Microbiol **73**(16): 5261-7.

40. Caporaso, J. G., K. Bittinger, et al. (2009). "PyNAST: a flexible tool for aligning sequences to a template alignment." Bioinformatics **26**(2): 266-7.

41. Price, M. N., P. S. Dehal, et al. (2009). "FastTree: computing large minimum evolution trees with profiles instead of a distance matrix." Mol Biol Evol **26**(7): 1641-50

42. Faith, D. P. (1992). "Conservation evaluation and phylogenetic diversity." Biological Conservation **61**(1): 1-10.

43. Lozupone, C. and R. Knight (2005). "UniFrac: a new phylogenetic method for comparing microbial communities." Appl Environ Microbiol **71**(12): 8228-35.

44. Tibshirani, R., T. Hastie, et al. (2002). "Diagnosis of multiple cancer types by shrunken centroids of gene expression." Proc Natl Acad Sci U S A **99**(10): 6567-72

17. APPENDICES

- Appendix A1 Site roster
- Appendix A2 Study contacts roster
- Appendix A3 Consent forms (current)
- Appendix A4 Test of comprehension (current)
- Appendix A5 Recruitment forms (draft)
- Appendix B Questionnaires (current)
- Appendix C Schedule of assessments
- Appendix D Manual of procedures (MOP)

17.1. Appendix A1: Site Roster

Site	Name	Address	Contact or comment
1	Lucile Packard Children's Hospital	725 Welch Road<br>Palo Alto, CA 94304	Natali Aziz, M.D.<br>(650) 721-1050<br>naziz@stanford.edu
2	Santa Clara Valley Medical Center Tully Road Clinic	500 Tully Rd<br>San Jose, CA 95111	Jenny Biller, M.D.<br>408-236-0176<br>Jenny.Biller@hhs.sccgov.org

17.2. Appendix A2: Study Contacts Roster

Name	Position	Email	Phone	Fax	Location
Aziz, Natalie	Co-investigator	naziz@stanford.edu	(650) 721-1050	(650) 723-7737	300 Pasteur Drive<br>Room HH333<br>Stanford, CA 94305
Biller, Jenny	Co-investigator	Jenny.Biller@hhs.sccgov<br>.org	408-236-0176		Valley Health Center<br>Bascom<br>750 S Bascom Ave<br>San Jose, CA 95128
Haggerty, Tom	<Lab>	tdhaggerty@gmail.com	(650) 725-7511	(650) 725-3584	Grant Building<br>Room S125<br>Stanford, CA 94305-5107
Ley, Catherine	Co-PI	cley@stanford.edu			HRP Redwood Building<br>T225<br>Stanford CA 94305
Parsonnet, Julie	PI	parsonnt@stanford.edu	(650) 725-4561	(650) 498-7011	Grant Building<br>Room S125 A<br>Stanford, CA 94305-5107
Sanchez, Luz	Study<br>Coordinator	mluz@stanford.edu	(650) 724-4947	(650) 725-3584	Grant Building<br>Room S131<br>Stanford, CA 94305-5107
IRB - Research<br>Compliance<br>Office					1501 S. California Avenue,<br>Palo Alto, CA 94304

17.3. Appendix A3: Consent Form (current)

See the following WORD documents:

- STORK Informed Consent (13Aug13)

17.4. Appendix A4: Test of Comprehension (current)

See the following .PDF document:

- STORK Comprehension Questionnaire 21Mar11

17.5. Appendix A5: Recruitment Forms

See the following WORD document:

- STORK Brochure 21Mar11

See the following .PDF documents:

- STORK Participant Screening Mar11
- STORK Personal Contact Form Mar11
- STORK Clinician Entry Form Mar11
- STORK Provider Authorization Form Mar11

17.6. Appendix B: Questionnaires (current)

See the associated documents:

17.6.1. Baseline (household visit)

17.6.2. Longitudinal, every 4 months (household visit)

17.6.3. Longitudinal, weekly (telephone or email)

- Mother
- Baby up to age 6 months
- Baby age 6-18 months
- Baby age 18-36 months

17.6.4. Chart abstraction

1. Mother

<u>Prenatal – Obstetric clinic chart</u>

For each visit, scheduled or non-scheduled

- Date
- Height
- Pre-pregnancy weight, weights over time (for total gain)
- Reproductive history
- Chronic conditions and associated treatments
- Diagnoses of infectious conditions and associated treatments
- Diagnoses of other conditions and associated treatments (e.g., gestational diabetes, etc.)

2. Child

<u>A. Delivery – Hospital chart</u>

- Date
- Sex
- Apgar scores
- Height
- Weight
- Complications (of or identified at delivery, birth defects, etc)

<u>B. Follow-up – Pediatrician's chart</u>

For each visit (well-baby or non-scheduled)

- Date
- Height
- Weight
- Feeding status (breast vs. formula, other, etc.)
- Developmental milestones
- Temperature (if assessed)

- Vaccinations performed
- Infectious disease diagnoses and associated treatments
- Other diagnoses and associated treatments

17.7. Appendix C: Schedule of Assessments

		Recruit-ment	Screening/ Enrollment (mother)	Prenatal period	Delivery	Months 1-11	Month 12	Months 13-23	Month 24	Months 25-35	Month 36 (or termination / last visit)	Questions/comments
**Administrative**												
	Review recruitment form and contact potential particpants	X										
	Obtain verbal informed consent for participation in study		X									
	Perform test of comprehension, get written consent and assign ID number		X									
	Administer inclusion and exclusion criteria		X									
	Collect/update locator information		X			@4, 8	X	@16, 20	X	@28, 32	X	
	Obtain signed consent for request for records from obstetrician (pregnancy) and from hospital (amnio, delivery)		X									
	Obtain signed consent for request for records/samples from hospital (delivery) and from pediatrician (baby visits)					X						Need to wait until first visit after birth as otherwise there will not be a medical record number
	Obtain random assignment		X									
	Obtain demographic information, family structure (household size, sibship structure, age, etc.), maternal and paternal weight and height by report.		X									
	Obtain change in family structure, child's participation in out of home care, dietary history/breast feeding and activity level, medical care.					@4, 8	X	@16, 20	X	@28, 32	X	
	Provide gift card					@4, 8	X	@16, 20	X	@28, 32	X	
	Provide stool/saliva collection kits					@4, 8	X	@16, 20	X	@28, 32		

		Recruit-ment	Screening/ Enrollment (mother)	Prenatal period	Delivery	Months 1-11	Month 12	Months 13-23	Month 24	Months 25-35	Month 36 (or termination / last visit)	Questions/comments
**"Weekly" reporting by mother**												
	Receive Maternal Infectious disease symptoms, antibiotic use			X								
	Receive Child Infectious diseases symptoms, clinic visit, antibiotic use, appetite					X (all weekly)	X	X	X	X	X	
**Biological samples / clinical measures**												
Maternal	Collect finger stick blood sample for microbial antibodies			X (26-30 wks) X (34-38 wks)								Samples collected during regular (scheduled) visits at Obstetric Clinic
Maternal	Collect, if obtained, amniotic fluid for microbial DNA extraction			X								
Maternal	Collect maternal stool/gingival samples for microbial DNA extraction		X				X		X		X	
Maternal	Collect maternal blood sample (for TNF, grehlin, insulin, leptin, IFN-gamma, IGF, glucagon, resistin, PAI-1, IL10, CRP, ESR, tricolsan); serum for microbial antibodies.		X				X		X		X	Baseline sample collected at regular visit at Obstetric Clinic
Maternal	Collect vaginal-rectal swab (routine visit)			X (36 wks)								
Maternal	Collect maternal urine sample (for tricolsan)		X				X		X		X	
Mother/Child	Collect cord blood (for antibody determination)				X							
Child	Temperature, weight					@4, 8	X	@16, 20	X	@28, 32	X	
Child	Child heel stick blood					@4, 8		@16, 20		@28, 32		
Child	Child urine					@4, 8	X	@16, 20	X	@28, 32	X	
Child	Child skin swab					@4, 8	X	@16, 20	X	@28, 32	X	

		Recruit-ment	Screening/ Enrollment (mother)	Prenatal period	Delivery	Months 1-11	Month 12	Months 13-23	Month 24	Months 25-35	Month 36 (or termination / last visit)	Questions/comments
Child	Child's blood for TNF, grehlin, insulin, leptin, IFN-gamma, IGF, glucagon, resistin, PAI-1, IL10, CRP, ESR , triclosan , LPS						X		X		X	
Child	Receive collection kits: Child stool/saliva samples for microbial flora					@4, 8	X	@16, 20	X	@28, 32	X	
House	Sample of dust under child's sleeping place, swab from the kitchen counter					@4, 8	X	@16, 20	X	@28, 32	X	
**Chart review**												
Mother	Medical record abstraction for infection, gestational diabetes, other illnesses, pre-pregnancy weight			X								
Child	Birth weight and height, Apgar scores				X							
Child	Abstracts of physician records for medical illnesses and treatments and height/weight as often as possible [from vaccination schedule at least], and any temperature, breast feeding and developmental milestones.					@6	X	@18	X	@30	X	
**Calorimetry**												
	Maternal and paternal resting metabolic rate					X (single reading)						
	Child's sleeping or resting metabolic rate through indirect calorimetry					@4		@16		@28		
**Triclosan accountability**												
	Evaluation of triclosan-containing cleaning products in the home			(Before baby)								
	Replacement of cleaning products			X		@4, 8	X	@16, 20	X	@28, 32	X	As needed else reviewed every 4 mo.

17.8. Appendix D: Manual of Procedures (MOP)

D1: Lab processing flow sheet

D2: Standard Operating Procedures for:

- Specimen collection
- Specimen processing
- Specimen shipment
- Specimen management
- Data management

D3: Protocols for:

- Collecting an infant's weight with a portable scale
- Collecting an armpit swab from an infant
- Collecting urine
- Collecting environmental samples from a kitchen counter
- Collecting environmental samples from under a crib
- Assessing resting metabolic rate in adults
- Assessing resting metabolic rate in infants and toddlers
- Taking a census of triclosan-containing products in the home
