# Supplementary material for: Triclosan and triclocarban exposure, infectious disease symptoms and antibiotic prescription in infants—A community-based randomized intervention
Source: PLoS One. 2018 Jun 28;13(6):e0199298. doi: 10.1371/journal.pone.0199298 (PMC6023107; doi:10.1371/journal.pone.0199298)
Supplement: S1 Text — (PDF) [file pone.0199298.s007.pdf]

S1 Appendix. Statistical model

We used mixed effects logistic regression techniques to test for differences in presence of infectious disease symptoms by intervention arm over time. We included linear and quadratic terms for days since randomization to allow for a potential non-linear relationship of illness over time among babies. The primary model for participant (i) over time (t) was:

$$\text{logit}(P(Y_{it}=1)) = \beta_0 + \beta_1 \text{ study\_arm} + \beta_2 \text{ time} + \beta_3 \text{ time}^2 + \beta_4 \text{ season} + \beta_5 \text{ study\_arm*time} + \beta_6 \text{ study\_arm*time}^2 + \gamma_i + \varepsilon_{it}$$

where Y_{it} was presence or absence of infectious disease symptoms for baby i at time t and γ_i was a random effect term for each baby to allow for within-baby correlation. We tested whether there was a difference between study arms in presence of infectious disease symptoms over time using a two-sided Likelihood ratio test with 2 degrees of freedom ($\beta_5 = \beta_6 = 0$) with a significance level of 0.05.

Specifically, we ran our analysis using the SAS procedure Proc Glimmix and specified an unstructured correlation structure. We ran a model with all terms listed above and a nested model excluding the interaction terms and conducted a likelihood ratio test to compare the two models.
