# Supplementary material for: Triclosan and triclocarban exposure, infectious disease symptoms and antibiotic prescription in infants—A community-based randomized intervention
Source: PLoS One. 2018 Jun 28;13(6):e0199298. doi: 10.1371/journal.pone.0199298 (PMC6023107; doi:10.1371/journal.pone.0199298)
Supplement: S2 Text — (PDF) [file pone.0199298.s008.pdf]

May 16, 2018

Analytic Datasets

Manuscript Title: Triclosan and triclocarban exposure, infectious disease symptoms and antibiotic prescription in infants – a community-based randomized intervention

Corresponding Author: Catherine Ley

There are 4 analytic datasets used for the results presented in this manuscript. All datasets are in .CSV format.

There is an excel spreadsheet titled 'S2_File2' which includes a data dictionary for each of the datasets listed below. The datasets are:

1) S2_File
   Contains subject-level data for all subjects participating in the randomized controlled trial. Includes variables for flags for inclusion in the mITT cohort and mPP cohort.

2) S3_File
   Contains survey-level data for mITT cohort for primary outcome analysis (has infection)

3) S4_File
   Contains survey-level data for mPP cohort for primary outcome analysis (has infection)

4) S5_File
   Contains visit-level data for antibiotic prescriptions based on medical record reviews
